# Palladium-Catalyzed Room Temperature Acylative Cross-Coupling of Activated Amides with Trialkylboranes

**DOI:** 10.3390/molecules23102412

**Published:** 2018-09-20

**Authors:** Weijia Shi, Gang Zou

**Affiliations:** School of Chemistry & Molecular Engineering, East China University of Science & Technology, 130 Meilong Rd, Shanghai 200237, China; 10111048@mail.ecust.edu.cn

**Keywords:** acylative cross-coupling, trialkylborane, amide activation, palladium, *N*-heterocyclic carbene

## Abstract

A highly efficient acylative cross-coupling of trialkylboranes with activated amides has been effected at room temperature to give the corresponding alkyl ketones in good to excellent yields by using 1,3-bis(2,6-diisopropyl)phenylimidazolylidene and 3-chloropyridine co-supported palladium chloride, the PEPPSI catalyst, in the presence of K_2_CO_3_ in methyl *tert*-butyl ether. The scope and limitations of the protocol were investigated, showing good tolerance of acyl, cyano, and ester functional groups in the amide counterpart while halo group competed via the classical Suzuki coupling. The trialkylboranes generated in situ by hydroboration of olefins with BH_3_ or 9-BBN performed similarly to those separately prepared, making this protocol more practical.

## 1. Introduction

Amides are unique and ubiquitous substructures in natural and artificial organic functional molecules, because the strong resonance between the carbonyl and amino groups leads to a highly inert and significantly planar linkage. However, the synthetic utility of amides as an acyl source had remained underdeveloped until the seminal publications in 2015 independently from Garg [1], Szostak [2], and ourselves [3], taking advantage of palladium or nickel catalysis for cleavage of electronically or sterically activated amide C-N bond, and formation of new carbon-carbon or carbon-oxygen bonds. In the past three years, many efforts have been made to expand the scope of amide counterparts, developing a variety of activated amides suitable for the acylative cross-coupling, e.g., *N*-acyl imides [4,5,6], *N*-Boc and *N*-Ts/Ms amides [7,8,9,10,11,12,13,14,15], *N*-acylsaccharins [16,17,18,19,20], and amides of heteroaromatic compounds [21,22]. Comparably, the carbon-centered nucleophile counterparts are still rather undeveloped, in particular, with respect to alkyl ones, although alkyl ketones have been widely found in biologically important molecules and synthetic building blocks for fine chemicals. In fact, besides the closely related esters [23,24,25], there are only two reports on the acylative cross-coupling of amides with alkyl reagents effected by using nickel catalysts. Garg and co-workers reported the first alkylation of amide derivatives by nickel-catalyzed acylative coupling with alkyl zinc reagents in 2016 [26]. Early this year, Rueping and co-workers described the other nickel-catalyzed cross-coupling of B-alkyl-9-BBN with amides [27]. After extension of the nucleophile counterparts from arylboronic acids to cost-effective diarylborinic acids and tetraarylboronates in palladium-catalyzed acylative cross-coupling of amides [28,29], we are interested in the reactivity of trialkylboranes, which could be readily prepared from alkyl Grignard reagents or olefins via hydroboration. Herein we report a highly efficient palladium-catalyzed acylative cross-coupling of activated amides with trialkylboranes at room temperature by using 1,3-bis(2,6-diisopropyl)phenylimidazolylidene (IPr) and 3-chloropyridine (3-ClPy) co-supported palladium chloride, the PEPPSI catalyst developed by Organ et al. [30].

## 2. Results and Discussion

**Scheme 1:**
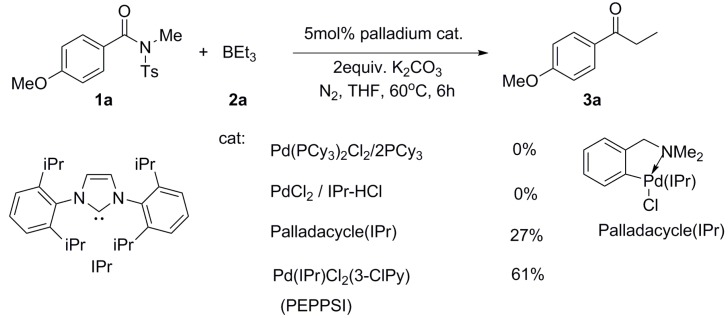
Screening of palladium catalyst for acyl alkylation of amides with alkylboranes.

Reaction of 4-methoxy-*N*-methyl-*N*-tosylbenzamide (**1a**) with the commercially available triethylborane (**2a**) (BEt_3_, 1M in THF) was chosen as the model to establish an optimal catalyst system for the acyl alkylation of activated amides (Scheme 1).

**Scheme 2:**
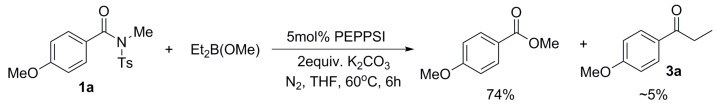
Unexpected acyl C-O cross-coupling of methyl diethylborinate.

The catalyst system Pd(PCy_3_)_2_Cl_2_/PCy_3_, which we previously established for acylative cross-coupling of amides with aryl boron compounds, was investigated, at first, in the presence of K_2_CO_3_ as base in THF. Unfortunately, palladium black developed immediately upon heating and no reaction was detected after 6h at 60 °C, indicating the incompatibility of the Pd(PCy_3_)_2_Cl_2_/PCy_3_ system with the high reducing ability of trialkylboranes. We then turned to the sterically demanding *N*-heterocyclic carbene (NHC) supported palladium catalysts pioneered by Nolan group [31], for example, 1,3-bis(2,6-diisopropylphenyl)imidazolium chloride (IPr-HCl)/palladium chloride (IPr-HCl/PdCl_2_) [32], IPr supported 2-((dimethylamino)methyl)phenyl palladium chloride (Palladacycle(IPr)) [33], and IPr and 3-chloropyridine co-supported PEPPSI catalyst (IPr)PdCl_2_(3-ClPy) [30]. Although the IPr-HCl/PdCl_2_ system also rapidly decomposed to palladium black and lost activity, the palladacycle/IPr and PEPPSI catalysts provided the desired cross-coupling product **3a** in 27% and 61% yields, respectively, from **1a** with 1.0 equiv **2a** in the presence of 2 equiv K_2_CO_3_. To test if dialkylboranes could be used in the acyl alkylation with amides, we also carried out the model reaction by using diethylborinate (Et_2_B(OMe), 1M in THF) under otherwise identical conditions (Scheme 2). Surprisingly, the C-O cross-coupling product, methyl 4-methoxybenzoate, was obtained in 74% yield, while the C-C coupling product **3a** was isolated in low (~5%) yield, implying a much slower transmetalation of alkyl group from boron to palladium than that of alkoxyl group in dialkylborinates.

To increase the yield of the desired C-C coupling product in the PEPPSI-catalyzed acyl alkylation of amides, the reaction conditions were optimized with respect to substrate ratio, solvent, base, and reaction temperature, etc. Bases proved to be crucial since no reaction took place in the absence of bases or in the presence of organic base, e.g., NEt_3_ and pyridine (Table 1, entries 2, 8 and 9). The stronger inorganic bases, Cs_2_CO_3_ (25%) and K_3_PO_4_ (33%), gave the lower yields of **3a** than that of K_2_CO_3_, albeit the reaction proceeded faster while the weaker ones, e.g., Na_2_CO_3_, NaOAc, and NaHCO_3_, resulted in recovering most of amide substrate **1a** (Table 1, entries 3–7). The yield of **3a** could be increased to 80% with 1.5 equiv BEt_3_ (Table 1, entry 10). Methyl *tert*-butyl ether (MTBE) appeared to be the choice of solvent, giving **3a** in 90% yield (Table 1, entry 12). The reaction occurred even at room temperature and an excellent yield (98%) for **3a** was obtained in 24 h (Table 1, entry 14). Given the advantages of room temperature organic synthesis [34], we reinvestigated the model reaction in THF or MTBE with K_2_CO_3_ or K_3_PO_4_ as the base (Table 1, entries 15–17). The results confirmed the best performance of the combination of MTBE with K_2_CO_3_.

Scope and limitations of the PEPPSI-catalyzed room temperature acylative cross-coupling of activated amides with trialkylboranes were briefly explored (Table 2). Influence of the amide structure was investigated at first under the optimized reaction conditions. Similar to tosyl-activated amide **1a**, mesyl (Ms, **1b**) analog also reacted efficiently to give **3a** in 98% yield, while *tert*-butyloxycarbonyl (Boc) activated amide (**1c**) showed comparably lower reactivity (75%) (Table 2, entries 1 and 2). Reaction of benzamides bearing an electron-withdrawing group, acyl (**1e**), cyano (**1f**), or ester (**1g**), at the *para*-position of the benzene ring gave the corresponding ketones (**3b**, **3c**, or **3d**) in 92%, 70%, or 91% yields, respectively, indicating good functional group compatibility of the PEPPSI-catalyzed acyl alkylation (Table 2, entries 4–6). When 4-chloro-*N*-methyl-*N*-tosylbenzamide (**1h**) was used as the substrate, the desired acylative cross-coupling product 1-(4-chlorophenyl)propanone was not obtained. Instead, double alkylation product 1-(4-ethylphenyl)propanone (**3e**) was isolated in 47% yield, which could be increased to 88% yield with 2.5 equiv BEt_3_ (Table 2, entry 7), similar to our previous investigation on the palladium-catalyzed acylative Suzuki coupling of arylboronic acids [3]. A small *ortho*-substituent at the benzene ring appeared to slightly hamper the coupling. In fact, 1-(*o*-tolyl)propan-1-one (**3f**) was isolated in 72% yield from *N*-methyl-*N*-tosyl-2-methylbenzamide (**1i**) (Table 2, entry 8). Alkyl amides (**1j** and **1k**) reacted similarly to give dialkyl ketones **3g** and **3h** in 95% and 98% yields, respectively (Table 2, entries 9 and 10).

Tri(*n*-butyl)borane (**2b**) reacted with **1a** similarly to triethylborane (**2a**) while no reaction of tricyclohexylborane was observed, implying the failure of transmetalation of *secondary*-alkyl group from boron to palladium. Hydroboration of alkenes represents an alternative route to primary alkyl boranes. However, the long-chain primary alkyl boranes prepared by hydroboration of alkenes are generally contaminated by the presence of branched-isomers due to the non-regiospecific addition of B-H to C-C double bonds [35]. Due to the inertness of the *secondary*-alkyl group in alkylboranes, reaction of **1a** with tri(octyl)boranes prepared by hydroboration of 1-octene proceeded comparably to that of tri(*n*-octyl)borane (**2c**) from Grignard reagent [36], giving **3j** in 91% and 99% yields, respectively (Table 2, entry 12). Given the fact that only one primary alkyl group of trialkylboranes is useful in the palladium-catalyzed acylative cross-coupling, it is more practical to use long-chain alkyl boranes prepared by hydroboration of corresponding olefins by dialkylborane, a more stable B-H source, e.g., 9-borabicyclo[3.3.1]nonane (9-BBN). Therefore, we further investigated use of B-octyl-9-BBN formed in situ from octene as the trialkylborane counterpart and obtained **3j** in 93% yield (Table 2, entry 13). *β*-Phenyl propiophenones, which show intrinsic deactivation of the lowest and thermally populated n, π*-triplet excited states in aryl alkyl ketones [37], could be readily obtained in 98% yield (**3k**) by the acyl alkylation of amide **1a** with B-(2-phenylpropyl)-9-BBN generated in situ from 9-BBN and α-methylstyrene. Alkyl and heterocyclic analogs, e.g., 1-cyclohexyl-3-phenylbutanone (**31**, 90%) and 3-phenyl-1-(thiophen-2-yl)butanone (**3m**, 97%) could also be prepared efficiently (Table 2, entries 14–16).

**Scheme 3:**
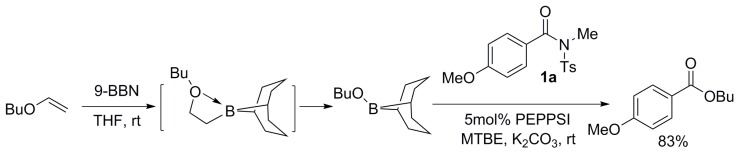
Formation of borinate and subsequent acyl C-O cross-coupling.

However, when amide **1a** was subjected to the hydroboration solution of *n*-butyl vinyl ether with 9-BBN the C-O coupling product (vide supra) butyl 4-methoxybenzoate, instead of the desired *β*-butoxy-4-methoxypropiophenone, was obtained in 83% yield, probably due to the facile *β*-alkoxy elimination of labile B-(2-butoxyethyl)-9-BBN via intramolecular O-B coordination to B-butoxy-9-BBN for the subsequent C-O cross-coupling (Scheme 3).

## 3. Materials and Methods

### 3.1. General

Chemicals obtained commercially were used as received. Nuclear magnetic resonance (NMR) spectra were recorded on a Bruker DPX-400 spectrometer (Bruker Co., Billerica, MA, USA) using residue of the deuterated solvent or tetramethylsilane (TMS) as the internal standard (Cambridge Isotope Lab. Inc., Tewksbury, MA, USA). For copies of ^1^H and ^13^C-NMR spectra of the products, please see Supplementary Materials. All products were isolated by flash chromatography using petroleum ether (Sinopharm Chemical Reagent Co. Ltd., Shanghai, China) (60–90 °C)/ethyl acetate (Sinopharm Chemical Reagent Co. Ltd., Shanghai, China) as eluents. Triethylborane (1 M in THF), tributylborane, 9-BBN (1 M in THF) and BH_3_ (1 M in THF) were purchased from J&K chemicals (Beijing, China). Amides [3], PEPPSI catalyst [30], 1,3-bis(2,6-diisopropylphenyl)imidazolium chloride (IPr-HCl) [32], palladacycle (IPr) [33], and tri(*n*-octyl)borane [36] were prepared according to the procedures reported previously. Solvents, methyl *tert*-butyl ether (MTBE), tetrahydrofuran (THF), and dioxane were dried over sodium while acetonitrile was distilled over CaH_2_ prior to use.

### 3.2. General Procedure for the PEPPSI-Catalyzed Cross-Coupling of N-Methyl-N-Tosylamides with Trialkylboranes

A Schlenk tube (20 mL) charged with amide (0.5 mmol), PEPPSI (0.025 mmol, 5 mol%), and K_2_CO_3_ (2 equiv) was degassed and then refilled with nitrogen, three times. Then, solvent MTBE (6.0 mL) was added via syringe followed by 0.75 mL (1.5 equiv) borane solution (1 M in THF), which was commercially available or was in situ prepared by hydroboration of olefin. The resulted mixture was stirred for 24 h at room temperature under N_2_ atmosphere. The reaction was quenched with iced water (5 mL) and extracted with MTBE (2 × 5 mL). The combined MTBE extracts were dried over anhydrous Na_2_SO_4_. After filtration, solvents were removed by rotavapor to afford the crude product, which was purified by flash column chromatography on silica gel using petroleum ether/EtOAc as the eluents.

*1-(4-Methoxyphenyl)propanone* (**3a**) [38]: Colorless oil (80.3 mg, 98%). ^1^H-NMR (400 MHz, CDCl_3_) δ(ppm): 7.91 (d, *J* = 8.8 Hz, 2H, Ar), 6.89 (d, *J* = 8.8 Hz, 2H, Ar), 3.82 (s, 3H, OMe), 2.91 (q, *J* = 7.2 Hz, 2H, CH_2_), 1.18 (t, *J* = 7.2 Hz, 3H, CH_3_). ^13^C-NMR (100 MHz, CDCl_3_) δ(ppm): 199.34 (C=O), 163.16 (C^4^_Ar_), 130.06 (C^2,6^_Ar_), 129.85 (C^1^_Ar_), 113.52 (C^3,5^_Ar_), 55.29 (OMe), 31.26 (CH_2_), 8.29 (CH_3_).

*1-(4-Acetylphenyl)propanone* (**3b**) [39]: White powder (80.9 mg, 92%), mp 69–71 °C. ^1^H-NMR (400 MHz, CDCl_3_) δ(ppm): 8.00 (s, 4H, Ar), 3.01 (q, *J* = 7.2 Hz, 2H, CH_2_), 2.62 (s, 3H, Ac), 1.21 (t, *J* = 7.2 Hz, 3H, CH_3_). ^13^C-NMR (100 MHz, CDCl_3_) δ(ppm): 200.13 (C=O), 197.46 (C=O), 139.96 (C^1/4^_Ar_), 139.90 (C^1/4^_Ar_), 128.41 (C_Ar_), 128.07 (C_Ar_), 32.15 (CH_2_), 26.81 (*Me*CO), 7.97 (CH_3_).

*4-Propionylbenzonitrile* (**3c**) [40]: White powder (55.8 mg, 70%), mp 53–55 °C. ^1^H-NMR (400 MHz, CDCl_3_) δ(ppm): 7.97 (d, *J* = 8.4 Hz, 2H, Ar), 7.69 (d, *J* = 8.4 Hz, 2H, Ar), 2.95 (q, *J* = 7.2 Hz, 2H, CH_2_), 1.15 (t, *J* = 7.2 Hz, 3H, CH_3_). ^13^C-NMR (100 MHz, CDCl_3_) δ(ppm): 199.16 (C=O), 139.64 (C^4^_Ar_), 132.33 (C_Ar_), 128.23 (C_Ar_), 117.84 (CN/C^1^_Ar_), 115.95 (CN/C^1^_Ar_), 32.01 (CH_2_), 7.75 (CH_3_).

*Methyl 4-propionylbenzoate* (**3d**) [41]: White crystalline powder, (87.1 mg, 91%), mp 81–83 °C. ^1^H-NMR (400 MHz, CDCl_3_) δ(ppm): 8.08 (d, *J* = 8.4 Hz, 2H, Ar), 7.97 (d, *J* = 8.4 Hz, 2H, Ar), 3.92 (s, 3H, OMe), 3.05 (q, *J* = 7.2 Hz, 2H, CH_2_), 1.21 (t, *J* = 7.2 Hz, 3H, CH_3_). ^13^C-NMR (100 MHz, CDCl_3_) δ(ppm): 200.15 (C=O), 166.17 (OC=O), 140.01 (C^1/4^_Ar_), 133.59 (C^1/4^_Ar_), 129.72 (C_Ar_), 127.78 (C_Ar_), 52.35 (OMe), 32.10 (CH_2_), 7.96 (CH_3_).

*1-(4-Ethylphenyl)propanone* (**3e**) [42]: Colorless oil (71.1 mg, 88%, with 2.5 equiv BEt_3_). ^1^H-NMR (400 MHz, CDCl_3_) δ(ppm): 7.81 (d, *J* = 8.4 Hz, 2H, Ar), 7.19 (d, *J* = 8.4 Hz, 2H, Ar), 2.90 (q, *J* = 7.2 Hz, 2H, COCH_2_), 2.61 (q, *J* = 7.6 Hz, 2H, ArCH_2_), 1.17 (t, *J* = 7.6 Hz, 3H, COCH_2_*CH*_3_), 1.13 (t, *J* = 7.2 Hz, 3H, Ar CH_2_*CH*_3_). ^13^C-NMR (100 MHz, CDCl_3_) δ(ppm): 200.51 (C=O), 149.71 (C^4^_Ar_), 134.59 (C^1^_Ar_), 128.14 (C_Ar_), 127.97 (C_Ar_), 31.60 (CO*CH*_2_), 28.85 (Ar*CH*_2_), 15.16 (ArCH_2_*CH*_3_), 8.27 (COCH_2_*CH*_3_).

*1-(o-Tolyl)propanone* (**3f**) [43]: Colorless oil (53.4 mg, 72%). ^1^H-NMR (400 MHz, CDCl_3_) δ(ppm): 7.53 (d, *J* = 7.6 Hz, 1H), 7.29–7.25 (m, 1H, Ar), 7.16–7.14 (m, 2H, Ar), 2.82 (q, *J* = 7.2 Hz, 2H, CH_2_), 2.41 (s, 3H, ArCH_3_), 1.11 (t, *J* = 7.2 Hz, 3H, CH_3_). ^13^C-NMR (100 MHz, CDCl_3_) δ(ppm): 205.04 (C=O), 138.01 (C^1/2^_Ar_), 137.76 (C^1/2^_Ar_), 131.81 (C_Ar_), 130.97 (C_Ar_), 128.19 (C_Ar_), 125.56 (C_Ar_), 34.64 (CH_2_), 21.19 (ArCH_3_), 8.31 (CH_3_).

*1-Phenylpentan-3-one* (**3g**) [44]: Colorless oil (77.1 mg, 95%). ^1^H-NMR (400 MHz, CDCl_3_) δ(ppm): 7.33–7.29 (m, 2H, Ph), 7.23–7.20 (m, 3H, Ph), 2.93 (t, *J* = 7.6 Hz, 2H, PhCH_2_*CH*_2_CO), 2.76 (t, *J* = 7.6 Hz, 2H, PhCH_2_), 2.43 (q, *J* = 7.2 Hz, 2H,CO *CH*_2_CH_3_), 1.07 (t, *J* = 7.2 Hz, 3H, CH_3_). ^13^C-NMR (100 MHz, CDCl_3_) δ(ppm): 210.61 (C=O), 141.10 (C^1^_Ph_), 128.41 (Ph), 128.24 (Ph), 126.00 (C^4^_Ph_), 43.81(PhCH_2_*CH*_2_CO), 36.05 (CO*CH*_2_CH_3_), 29.77 (PhCH_2_), 7.69 (CH_3_).

*1-(4-Methoxyphenyl)pentan-3-one* (**3h**) [45]: Colorless oil (94.3 mg, 98%). ^1^H-NMR (400 MHz, CDCl_3_) δ(ppm): 7.09 (d, *J* = 8.8 Hz, 2H, Ar), 6.81 (d, *J* = 8.8 Hz, 2H, Ar), 3.77 (s, 3H, OMe), 2.84 (t, *J* = 7.6 Hz, 2H, PhCH_2_*CH*_2_CO), 2.64 (t, *J* = 7.6 Hz, 2H, PhCH_2_), 2.39 (q, *J* = 7.6 Hz, 2H, CO*CH*_2_CH_3_), 1.03 (t, *J* = 7.6 Hz, 3H, CH_3_). ^13^C-NMR (100 MHz, CDCl_3_) δ(ppm): 210.80 (C=O), 157.85 (C^4^_Ar_), 133.12 (C^1^_Ar_), 129.16 (C_Ar_), 113.79 (C_Ar_), 55.17 (OMe), 44.09 (PhCH_2_*CH*_2_CO), 36.08 (CO*CH*_2_CH_3_), 28.93 (PhCH_2_), 7.68 (CH_3_).

*1-(4-Methoxyphenyl)pentanone* (**3i**) [46]: Colorless oil (93.5 mg, 97%). ^1^H-NMR (400 MHz, CDCl_3_) δ(ppm): 7.93 (d, *J* = 8.8 Hz, 2H, Ar), 6.91 (d, *J* = 9.2 Hz, 2H, Ar), 3.85 (s, 3H, OMe), 2.90 (t, *J* = 7.6 Hz, 2H, COCH_2_), 1.73–1.66 (m, 2H, COCH_2_*CH*_2_), 1.44–1.34 (m, 2H, COCH_2_CH_2_*CH*_2_), 0.94 (t, *J* = 7.6 Hz, 3H). ^13^C-NMR (100 MHz, CDCl_3_) δ(ppm): 199.20 (C=O), 163.23 (C^4^_Ar_), 130.25 (C^2,6^_Ar_), 130.11(C^1^_Ar_), 113.59 (C^3,5^_Ar_), 55.37 (OMe), 37.95 (COCH_2_), 26.68 (CH_2_), 22.48 (CH_2_), 13.90 (CH_3_).

*1-(4-Methoxyphenyl)nonanone* (**3j**) [47]: Colorless oil (123.0 mg, 99%, with tri(*n*-octyl)borane; 115 mg, 93% with hydroboration of octene with 9-BBN; 113 mg, 91% with hydroboration of octene with BH_3_. ^1^H-NMR (400 MHz, CDCl_3_) δ(ppm): 7.86 (d, *J* = 9.2 Hz, 2H, Ar), 6.84 (d, *J* = 9.2 Hz, 2H, Ar), 3.78 (s, 3H, OMe), 2.82 (t, *J* = 7.6 Hz, 2H, COCH_2_), 1.63 (t, *J* = 7.6 Hz, 2H, COCH_2_*CH*_2_), 1.29–1.19 (m, 10H, (CH_2_)_5_), 0.80 (t, *J* = 7.2Hz, 3H, CH_3_). ^13^C-NMR (100 MHz, CDCl_3_) δ(ppm): 199.20 (C=O), 163.23 (C^4^_Ar_), 130.25 (C^2,6^_Ar_), 130.13 (C^1^_Ar_), 113.60 (C^3,5^_Ar_), 55.37 (OMe), 38.26 (COCH_2_), 31.79 (CH_2_), 29.41 (CH_2_), 29.39 (CH_2_), 29.14 (CH_2_), 24.59 (CH_2_), 22.61 (CH_2_), 14.05 (CH_3_).

*1-(4-Methoxyphenyl)-3-phenylbutanone* (**3k**) [46]: White powder (125.0 mg, 98%), mp 84–86 °C. ^1^H-NMR (400 MHz, CDCl_3_) δ(ppm): 7.93 (d, J = 8.8 Hz, 2H, Ar), 7.34–7.30 (m, 4H, Ph), 7.23–7.19 (m, 1H, Ph), 6.92 (d, *J* = 8.8 Hz, 2H, Ar), 3.86 (s, 3H, OMe), 3.55–3.47 (m, 1H, CH), 3.26 (dd, *J*_1_ = 16.4 Hz, *J*_2_ = 5.6 Hz, 1H, CH_2_), 3.14 (dd, *J*_1_ = 16.0 Hz, *J*_2_ = 8.4 Hz, 1H, CH_2_), 1.34 (d, *J* = 7.8, 3H, CH_3_). ^13^C-NMR (100 MHz, CDCl_3_) δ(ppm): 197.59 (C=O), 163.31 (C^4^_Ar_), 146.65 (C^1^_Ph_), 130.28 (C_Ar_/C_Ph_), 130.21 (C^1^_Ar_), 128.43 (C_Ph_), 126.79 (C_Ph_), 126.15 (C^4^_Ph_), 113.61 (C^3,5^_Ar_), 55.37 (OMe), 46.59 (COCH_2_), 35.67 (CH), 21.79 (CH_3_).

*1-Cyclohexyl-3-phenylbutanone* (**3l**): Colorless oil (103.7 mg, 90%). ^1^H-NMR (400 MHz, CDCl_3_) δ(ppm): 7.33–7.29 (m, 2H, Ph), 7.25–7.19 (m, 3H, Ph), 3.41–3.32 (m, 1H, PhCH), 2.77 (dd, *J*_1_ = 16.8 Hz, *J*_2_ = 6.4 Hz, 1H,CO CH), 2.69 (dd, *J*_1_ = 16.4 Hz, *J*_2_ = 8.0 Hz, 1H, COCH_2_), 2.29–2.22 (m, 1H, COCH), 1.83–1.65 (m, 5H, Cy), 1.35–1.17 (m, 8H, Cy overlapped with CH_3_). ^13^C-NMR (100 MHz, CDCl_3_) δ(ppm): 212.83 (C=O), 146.54 (C^1^_Ph_), 128.39 (C_Ph_), 126.75 (C_Ph_), 126.11 (C^4^_Ph_), 51.19 (CO*CH*), 49.10 (CO*CH*_2_), 35.02 (CH), 28.22 (Cy), 28.05 (Cy), 25.78 (Cy), 25.61(Cy), 25.53 (Cy), 21.79 (CH_3_). HRMS (ESI) *m*/*z* calcd for C_16_H_22_ONa [M + Na]^+^ 253.1568, found 253.1569.

*3-Phenyl-1-(thiophen-2-yl)butanone* (**3m**) [48]: Colorless oil (111.5 mg, 97%). ^1^H-NMR (400 MHz, CDCl_3_) δ(ppm): 7.70–7.69 (m, 1H, thiophenyl), 7.64–7.63 (m, 1H, thiophenyl), 7.36–7.28 (m, 4H, Ph), 7.25–7.23 (m, 1H, Ph), 7.14–7.12 (m, 1H, thiophenyl), 3.56–3.51 (m, 1H, PhCH), 3.24 (dd, *J*_1_ = 16.0 Hz, *J*_2_ = 6.0 Hz, 1H, CH_2_CO), 3.14 (dd, *J*_1_ = 15.6 Hz, *J*_2_ = 8.4 Hz, 1H, CH_2_CO), 1.38 (d, *J* = 6.8 Hz, 3H, CH_3_). ^13^C-NMR (100 MHz, CDCl_3_) δ(ppm): 191.94 (C=O), 146.18 (C^1^_Ph_), 144.60 (C^2^ thiophenyl), 133.56 (thiophenyl), 131.81 (thiophenyl), 128.49(C_Ph_), 128.01(thiophenyl), 126.78 (C_Ph_), 126.30 (C^4^_Ph_), 47.78 (COCH_2_), 35.93 (CH), 21.64 (CH_3_).

*1-Phenylnonanone* (**3n**) [47]: Colorless oil (106.8 mg, 98%). ^1^H-NMR (400 MHz, CDCl_3_) δ(ppm): 7.97–7.95 (m, 2H, Ph), 7.57–7.53 (m, 1H, Ph), 7.47–7.44 (m, 2H, Ph), 2.96 (t, *J* = 7.6 Hz, 2H, COCH_2_), 1.77–1.69 (m, 2H, COCH_2_*CH*_2_), 1.39–1.25 (m, 10H, (CH_2_)_5_), 0.88 (t, *J* = 6.8 Hz, 3H, CH_3_). ^13^C-NMR (100 MHz, CDCl_3_) δ(ppm): 200.62 (C=O), 137.05 C^1^_Ph_), 132.83 (C^4^_Ph_), 128.51 (C_Ph_), 128.02 (C_Ph_), 38.61 (CO*CH*_2_), 31.81 (CH_2_), 29.42 (CH_2_), 29.36 (CH_2_), 29.15 (CH_2_), 24.36 (CH_2_), 22.63 (CH_2_), 14.08 (CH_3_).

## 4. Conclusions

In summary, we have developed a highly efficient acylative cross-coupling of trialkylboranes with activated amides by using 1,3-bis(2,6-diisopropyl)phenylimidazolylidene and 3-chloropyridine co-supported palladium chloride, the PEPPSI catalyst, under mild conditions. Bases appeared to play a key role in the reaction, among which K_2_CO_3_ performed best in MTBE at room temperature. The reaction proceeded to give alkyl ketones in good to excellent yields, tolerating a variety of functional groups in the amide counterpart. Unlike the high-order arylboron compounds, in which all the aryl groups react effectively, only one of the three primary alkyl groups in trialkylboranes could be used as alkyl source for the acyl alkylation. The trialkylboranes generated in situ by hydroboration of olefins with BH_3_ or 9-BBN performed comparably to those separately prepared. This protocol complements ketone synthesis via palladium-catalyzed acylative cross-coupling of amides, providing a feasible access to both monoalkyl and dialkyl ketones.

## Figures and Tables

**Table 1:**
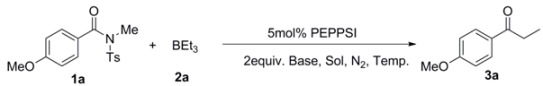
PEPPSI-catalyzed acyl alkylation reaction of amide **1a** with triethylborane. ^a^

Entry	Equiv of 2a	Solvent	Base ^b^	T (°C)	Time (h)	Yield (%) ^c^
1	1.1	THF	K_2_CO_3_	60	5	61
2	1.1	THF	/	60	5	NR ^d^
3	1.1	THF	Cs_2_CO_3_	60	5	25
4	1.1	THF	K_3_PO_4_	60	5	33
5	1.1	THF	Na_2_CO_3_	60	8	15
6	1.1	THF	NaHCO_3_	60	8	Trace ^d^
7	1.1	THF	NaOAc	60	8	Trace ^d^
8	1.1	THF	Et_3_N	60	8	NR ^d^
9	1.1	THF	Pyridine	60	8	NR ^d^
10	1.5	THF	K_2_CO_3_	60	5	80
11	1.5	Dioxane	K_2_CO_3_	60	8	35
12	1.5	MTBE	K_2_CO_3_	55	8	90
13	1.5	MeCN	K_2_CO_3_	60	8	17
14	1.5	MTBE	K_2_CO_3_	rt	24	98
15	1.5	MTBE	K_3_PO_4_	rt	24	84
16	1.5	THF	K_2_CO_3_	rt	24	82
17	1.5	THF	K_3_PO_4_	rt	12	40

^a^ Reaction was run at 0.5 mol scale with respect to **1a**; ^b^ 2 equiv base used but no base used in Entry 2; ^c^ Isolated yield; ^d^ Amide **1a** recovered.

**Table 2:**
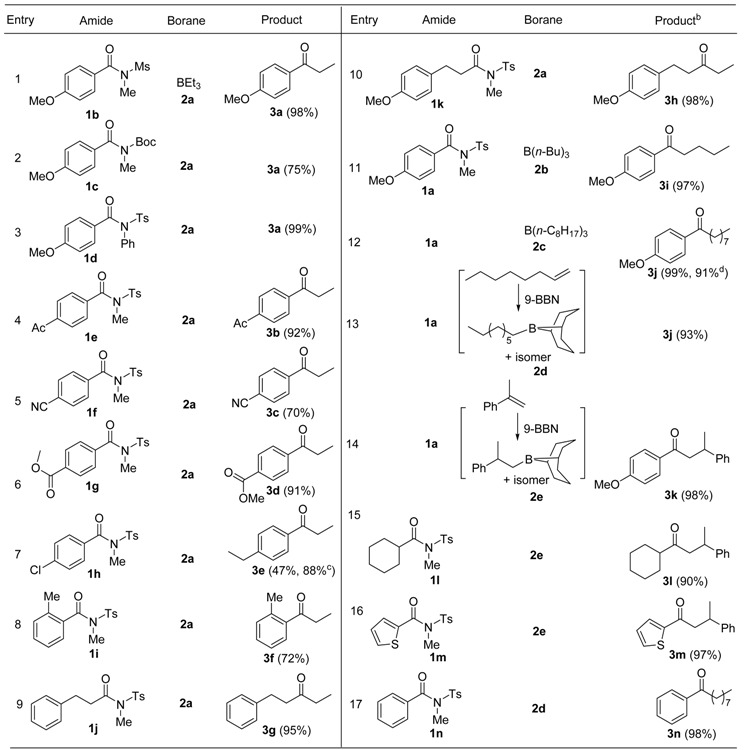
Scope and limitations of the PEPPSI-catalyzed acyl alkylation reaction of amides with trialkylboranes ^a^.

^a^ Reaction was run at 0.5 mol scale with respect to amides with 1.5 equiv trialkylboranes; ^b^ Isolated yield; ^c^ With 2.5 equiv BEt_3_; ^d^ B(*n*-C_8_H_17_)_3_ and branched isomers in situ generated from hydroboration of octene with 1.0 equiv BH_3_ in THF at room temperature was used.

## Supplementary Materials

The following are available online, Figures S1–S28: ^1^H and ^13^C-NMR of products **3a**–**3n**, Figure S29 HRMS of **3l**.
