# Supplementary material for: Palladium-Catalyzed Room Temperature Acylative Cross-Coupling of Activated Amides with Trialkylboranes
Source: Molecules. 2018 Sep 20;23(10):2412. doi: 10.3390/molecules23102412 (PMC6222728; doi:10.3390/molecules23102412)
Supplement: Supplementary file 1 [file molecules-23-02412-s001.pdf]

Supplementary Materials

Palladium-Catalyzed Room Temperature Acylative Cross-Coupling of Activated Amides with Trialkylboranes

Weijia Shi and Gang Zou

School of Chemistry & Molecular Engineering, East China University of Science & Technology, 130 Meilong Rd, Shanghai, China

Copies of ^{1}H NMR, ^{13}C NMR **3a-3n** and HRMS Spectrum of **3l**

Figure S1. ^{1}H NMR of 1-(4-methoxyphenyl)propanone (**3a**)

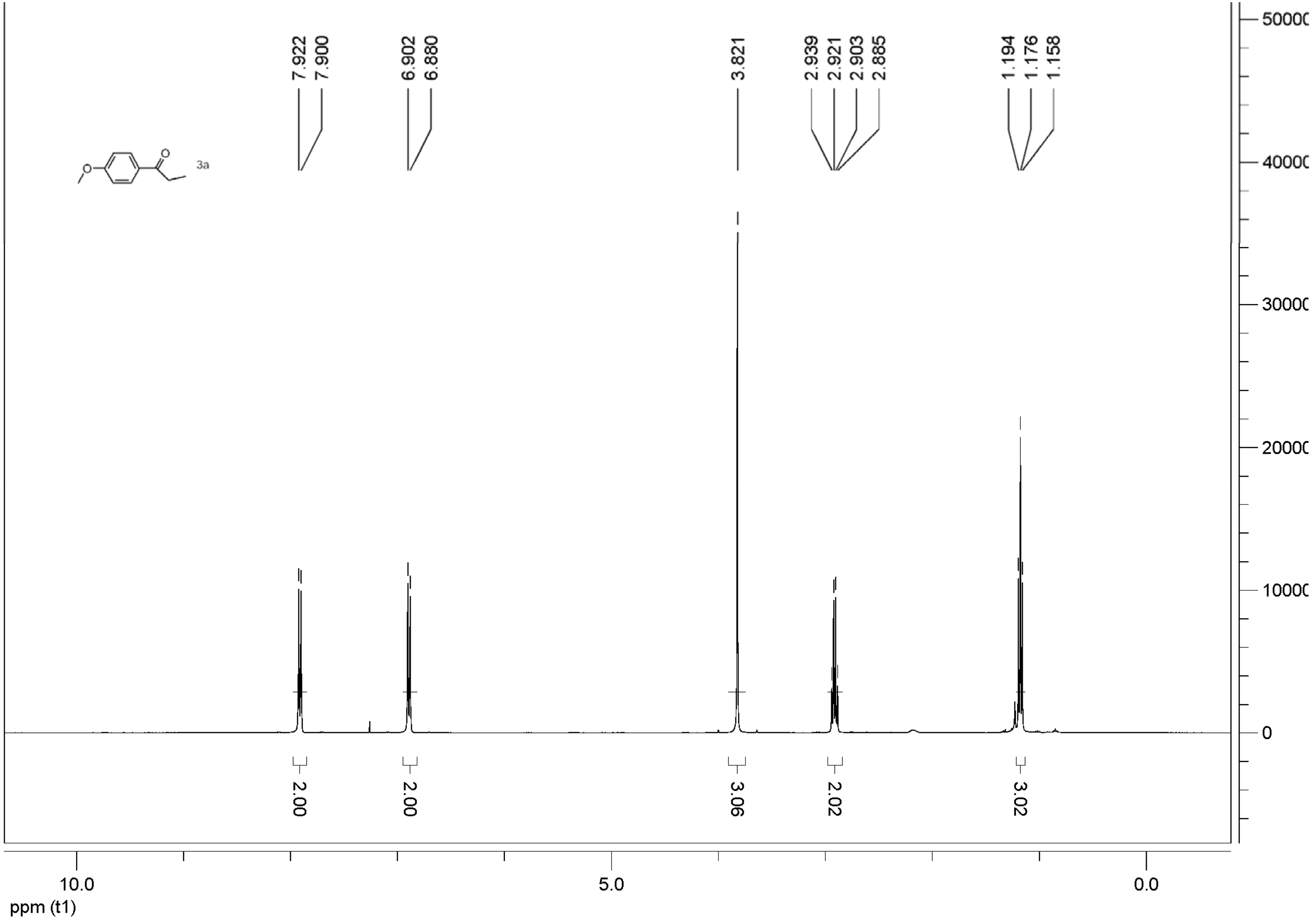

Figure S2. ^{13}C NMR of 1-(4-methoxyphenyl)propanone (**3a**)

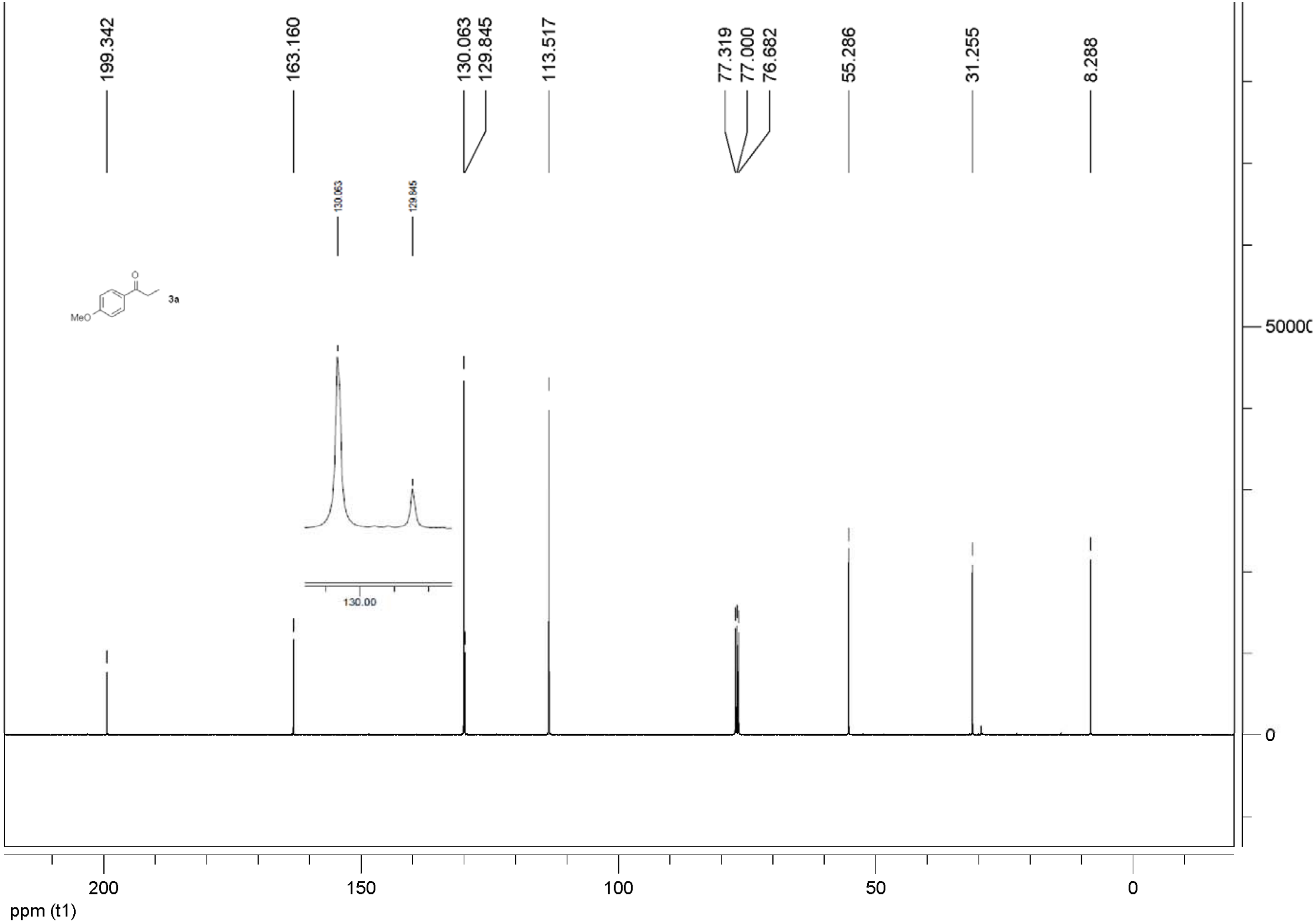

Figure S3. ^{1}H NMR of 1-(4-acetylphenyl)propanone (**3b**)

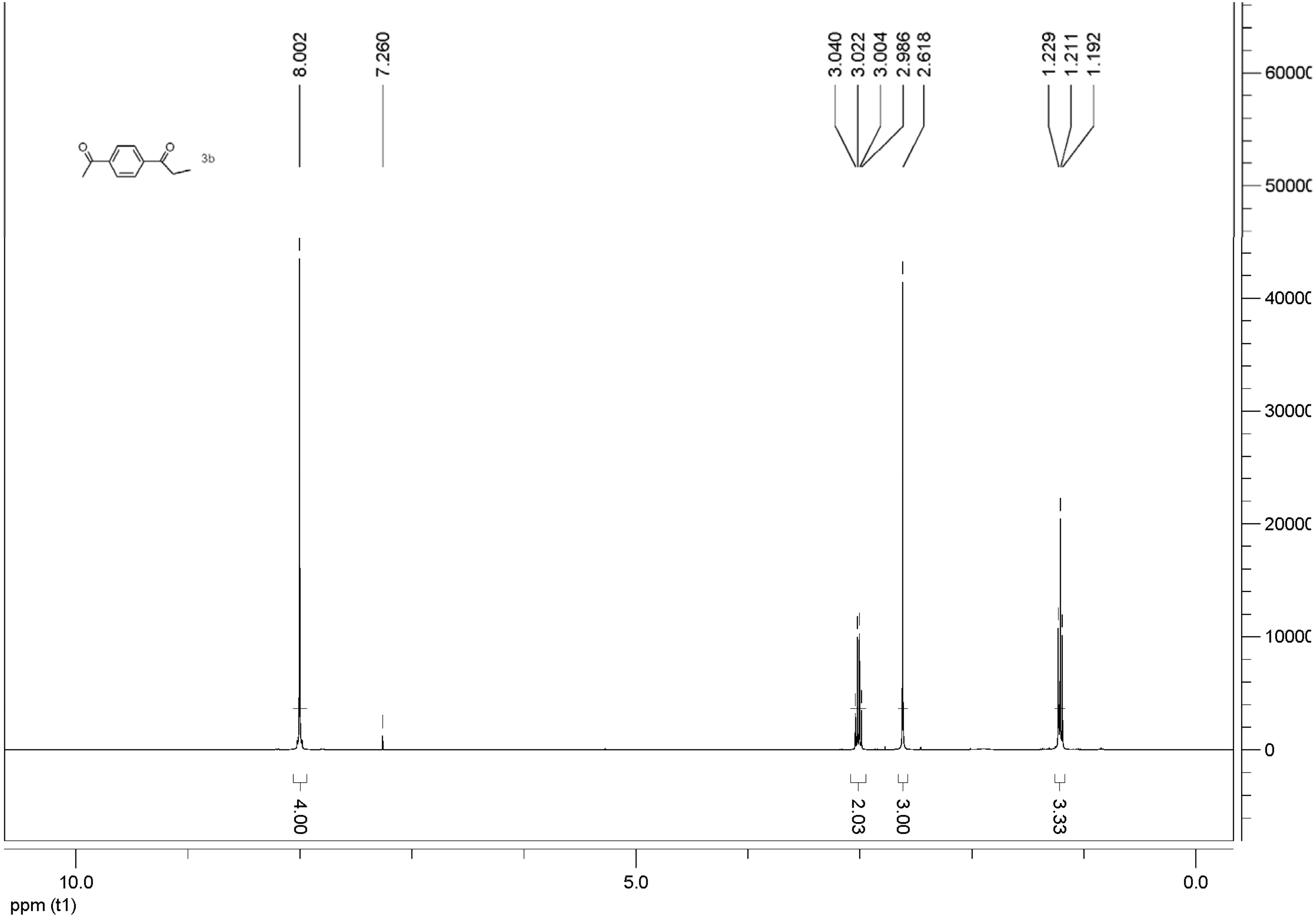

Figure S4. ^{13}C NMR of 1-(4-acetylphenyl)propanone (**3b**)

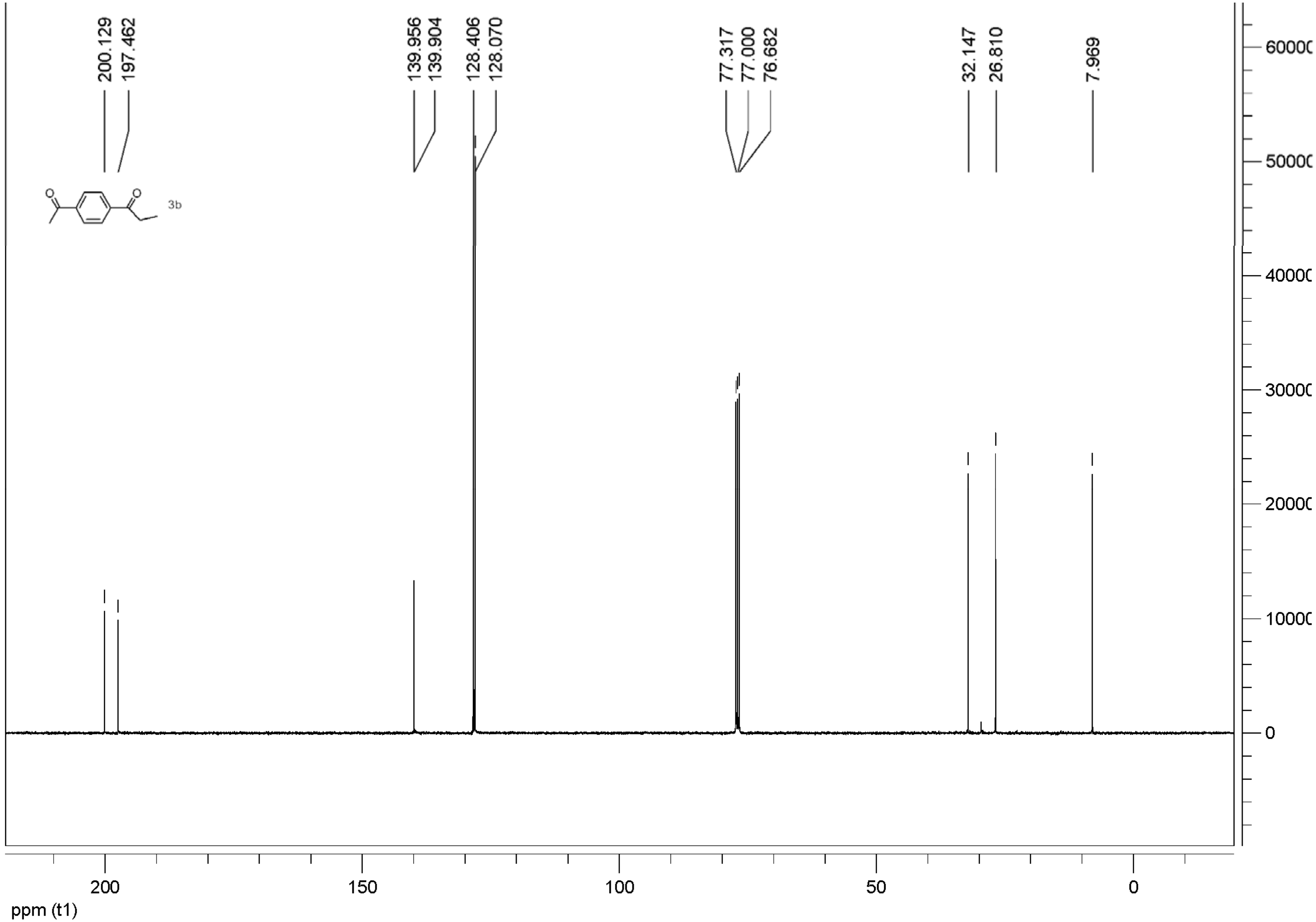

Figure S5. ¹H NMR of 4-propionylbenzonitrile (**3c**)

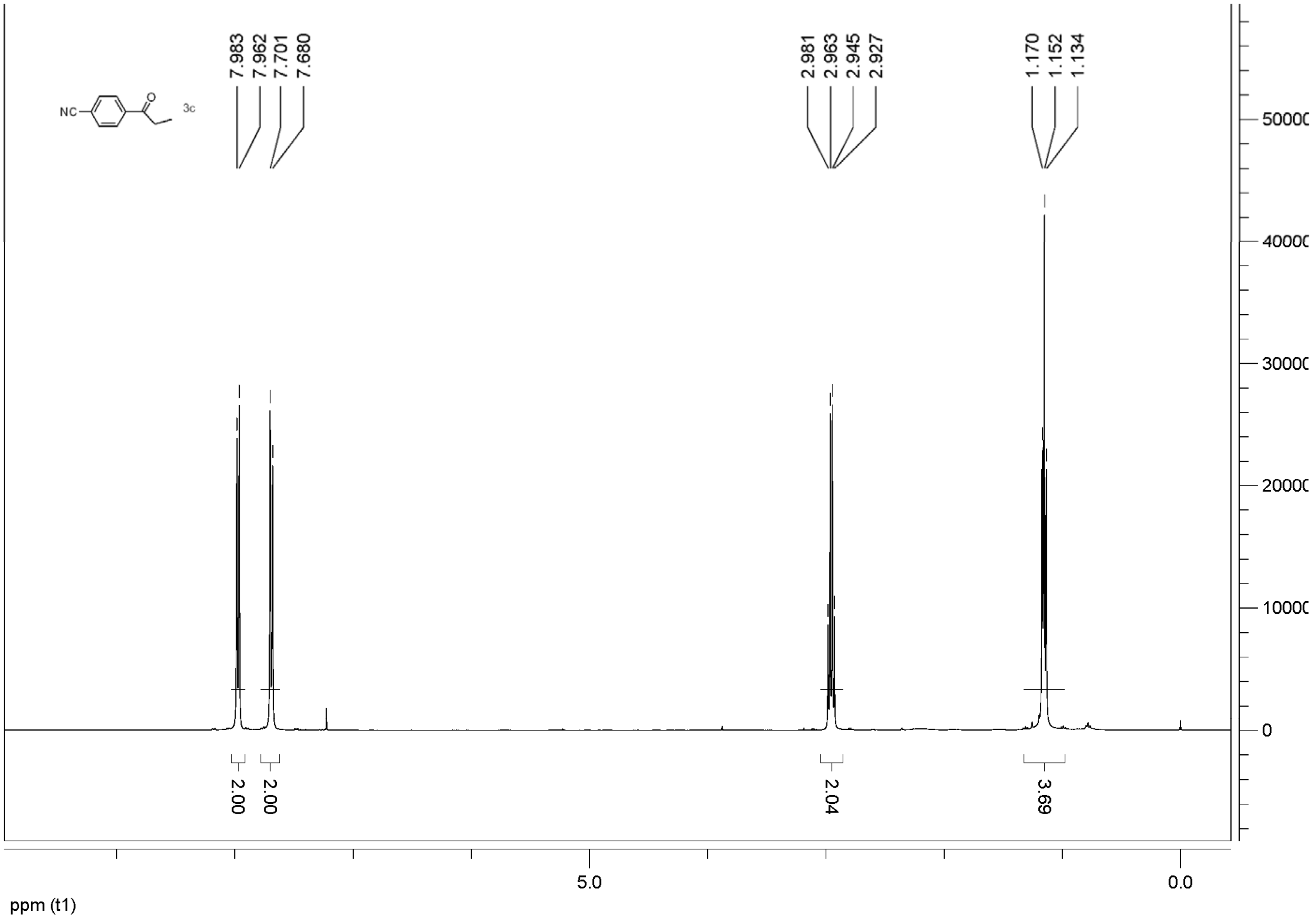

Figure S6. ^{13}C NMR of 4-propionylbenzonitrile (**3c**)

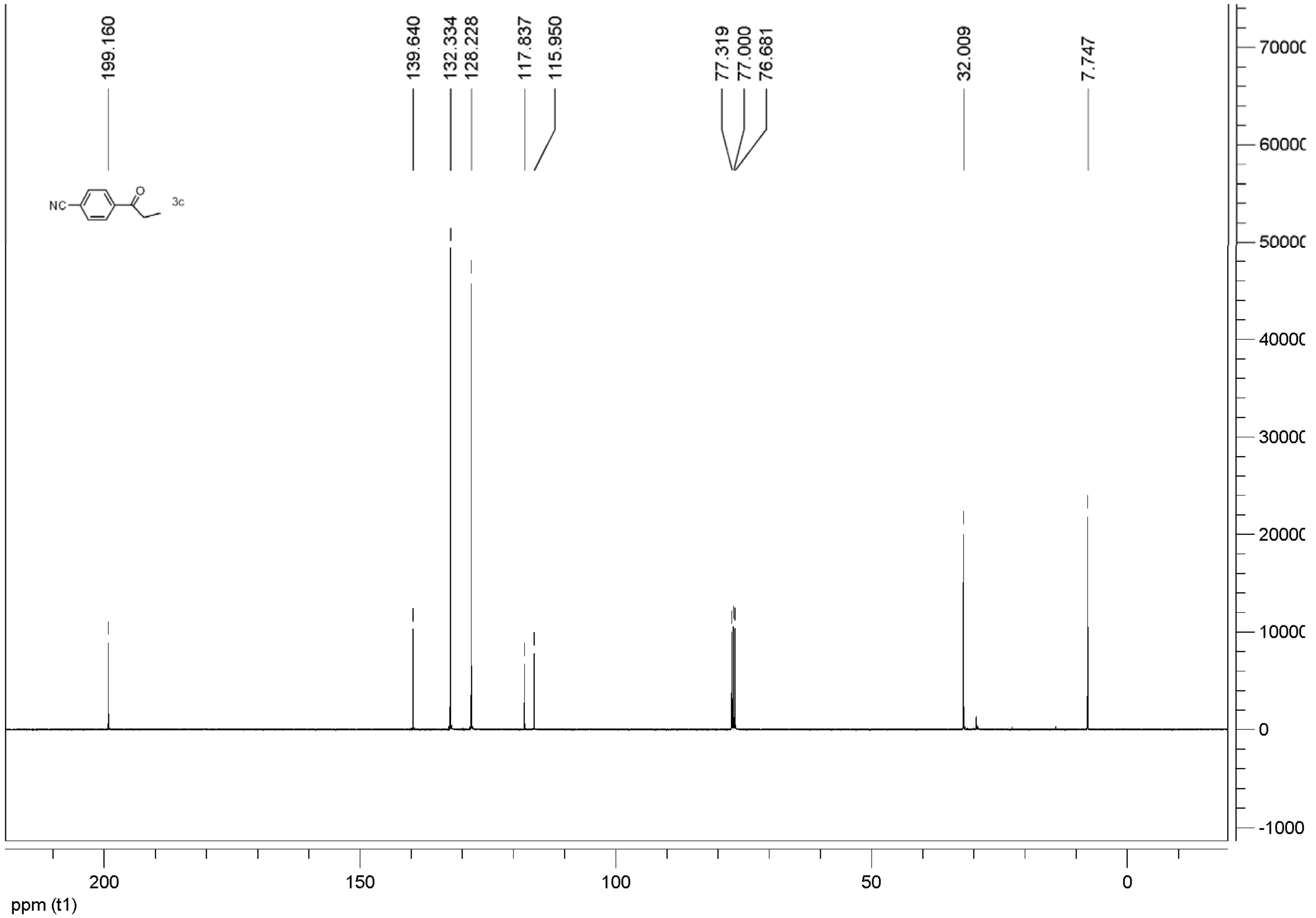

Figure S7. ^{1}H NMR of methyl 4-propionylbenzoate (**3d**)

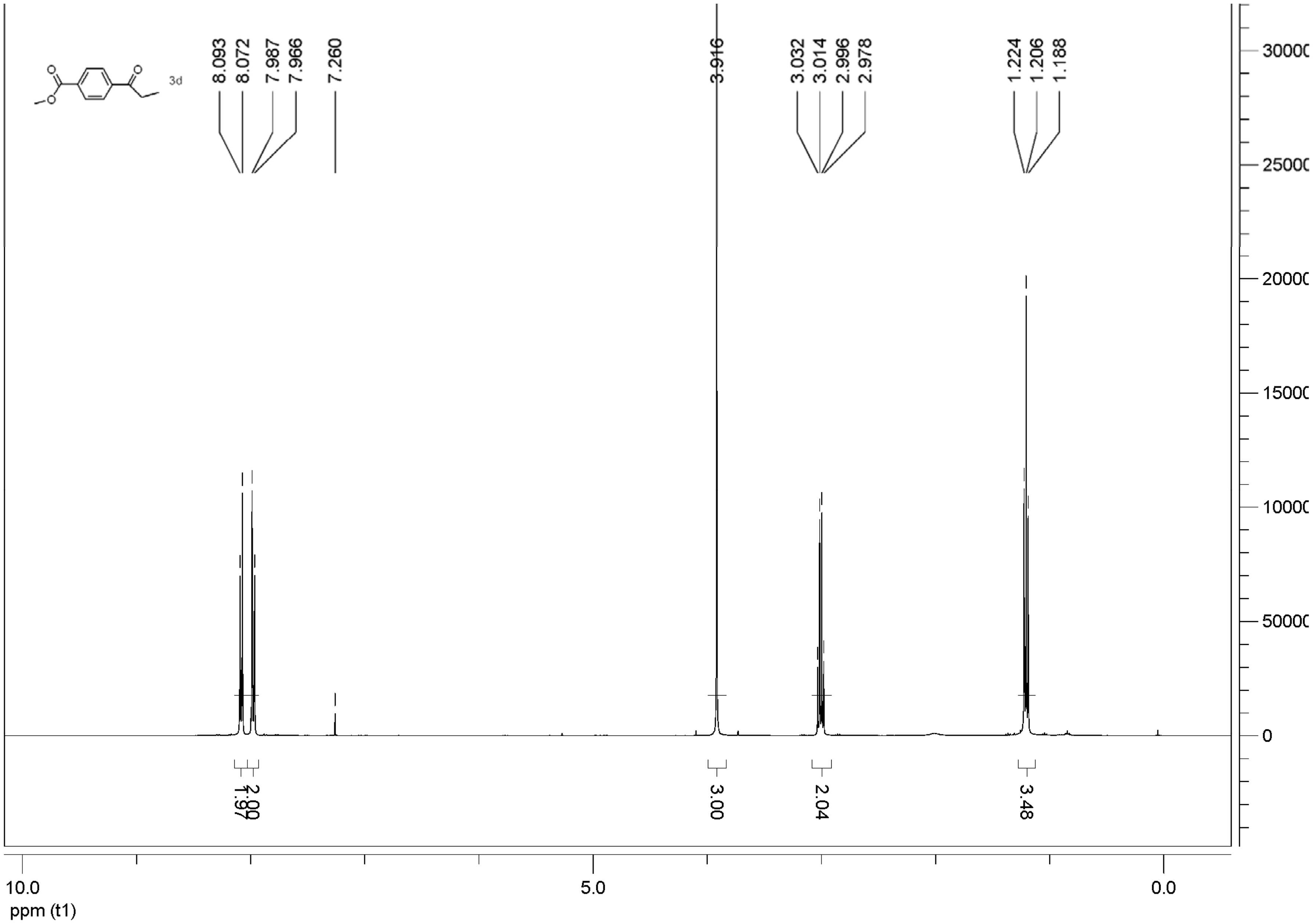

Figure S8. ^{13}C NMR of methyl 4-propionylbenzoate (**3d**)

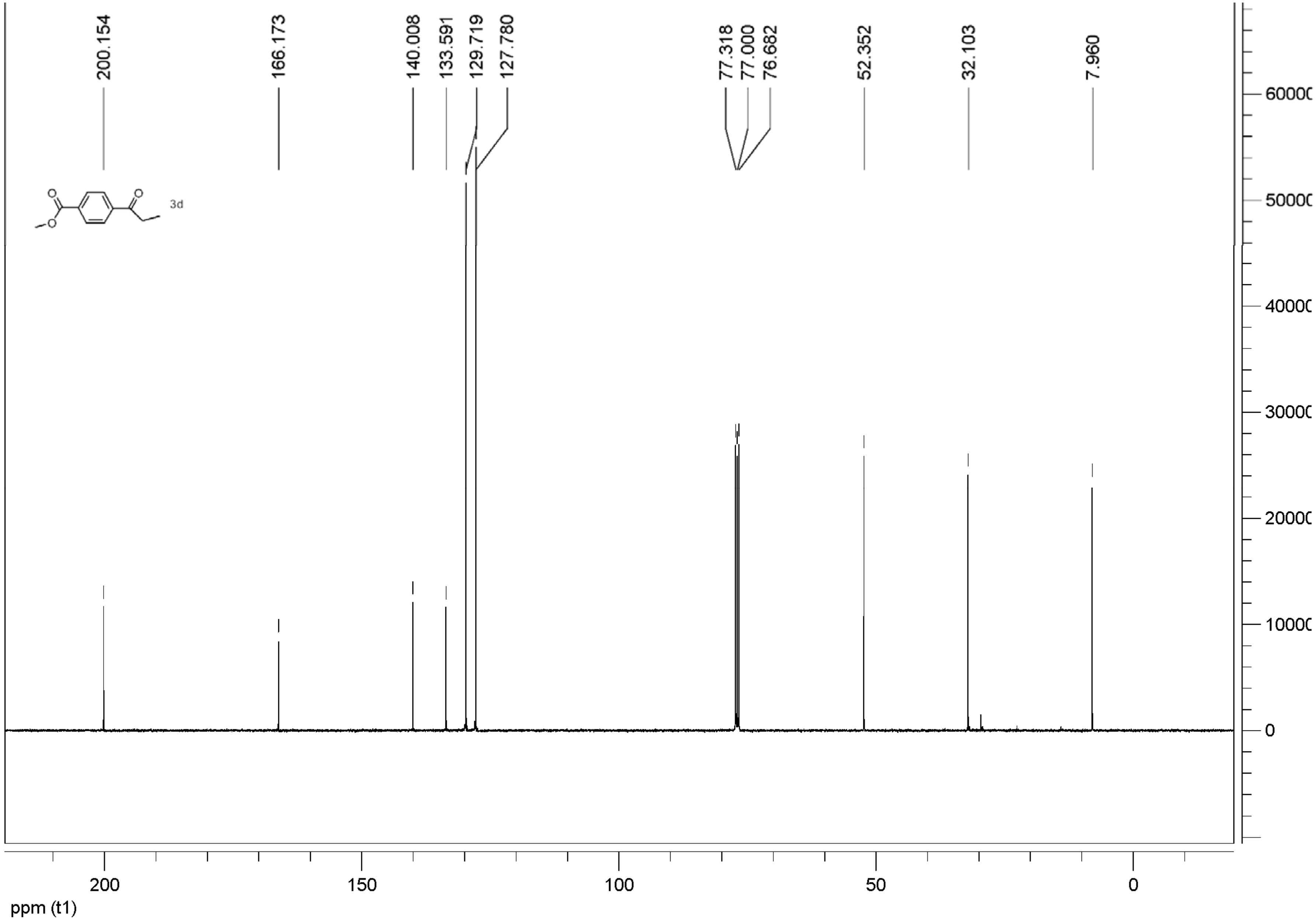

Figure S9. ^{1}H NMR of 1-(4-ethylphenyl)propanone (**3e**)

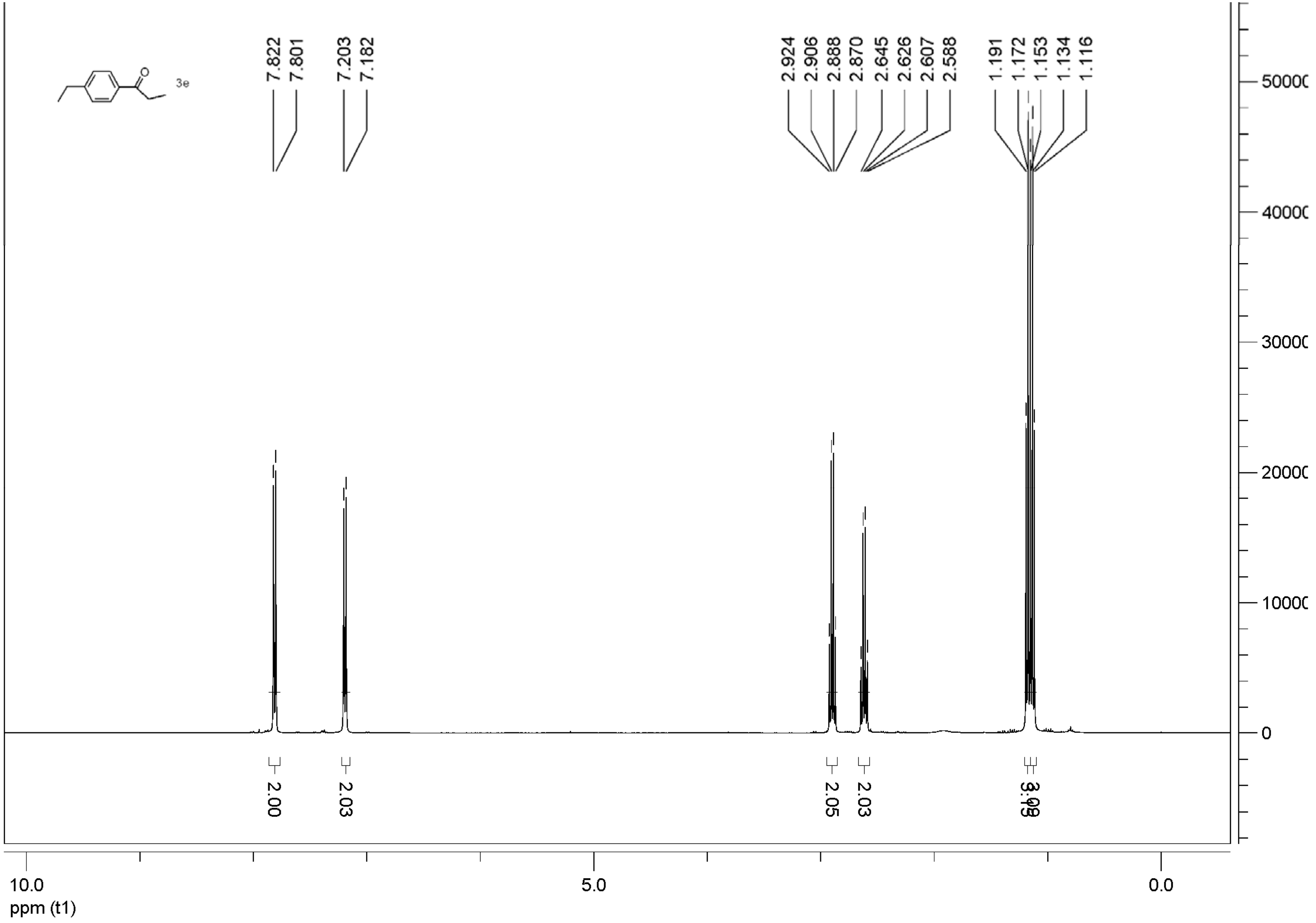

Figure S10. ^{13}C NMR of 1-(4-ethylphenyl)propanone (**3e**)

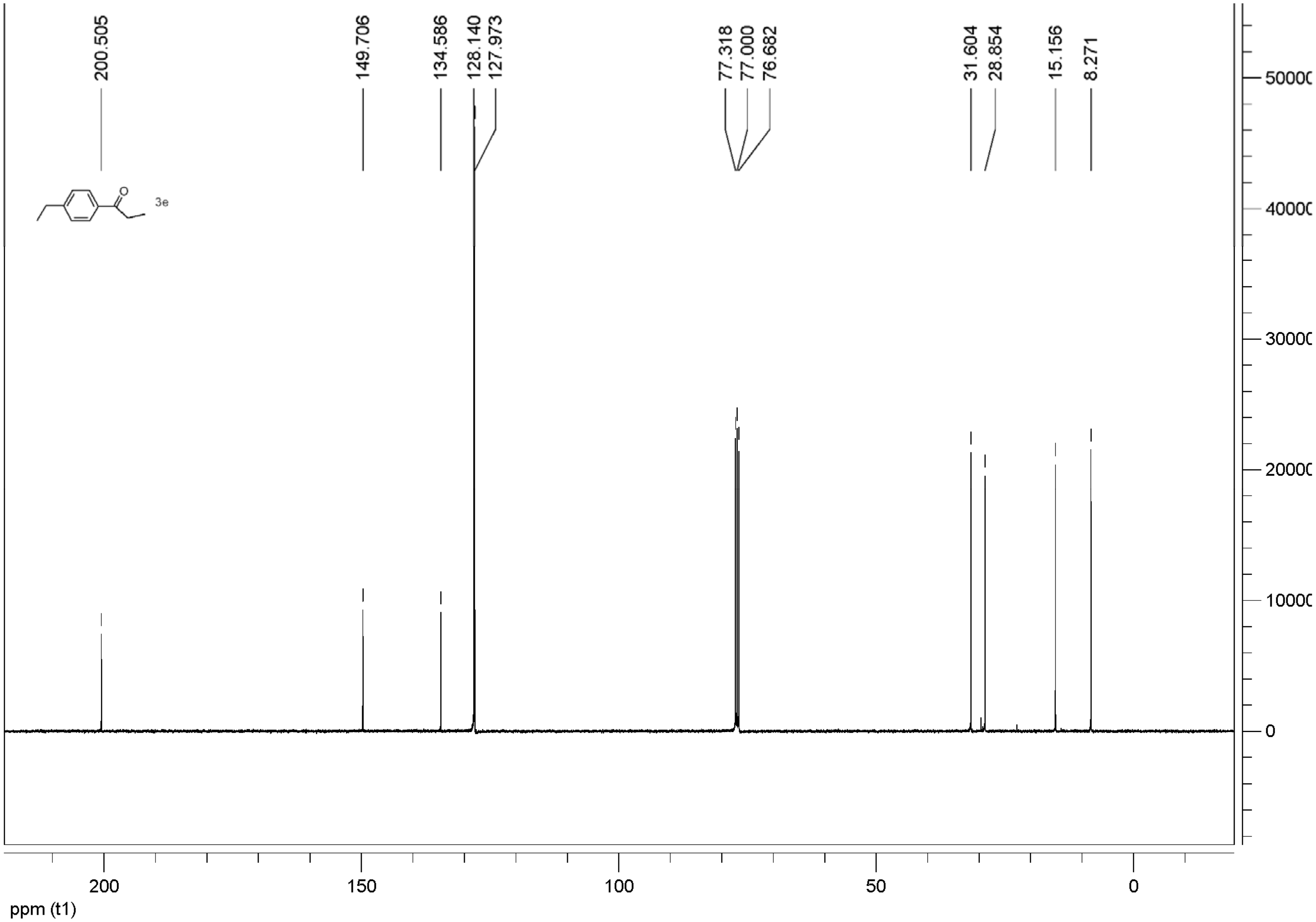

Figure S11. ^{1}H NMR of 1-(o-tolyl)propanone (**3f**)

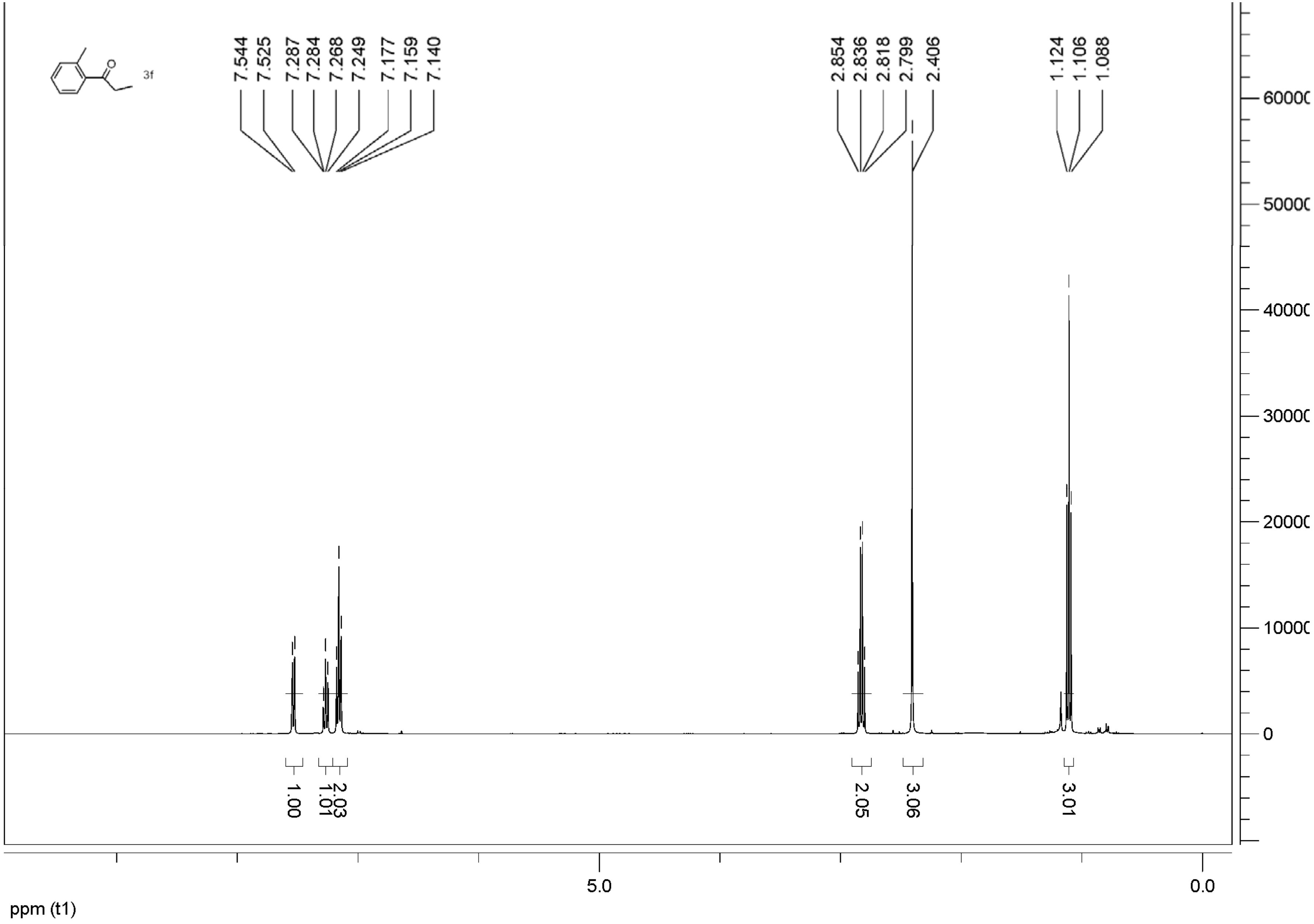

Figure S12. ^{13}C NMR of 1-(o-tolyl)propanone (**3f**)

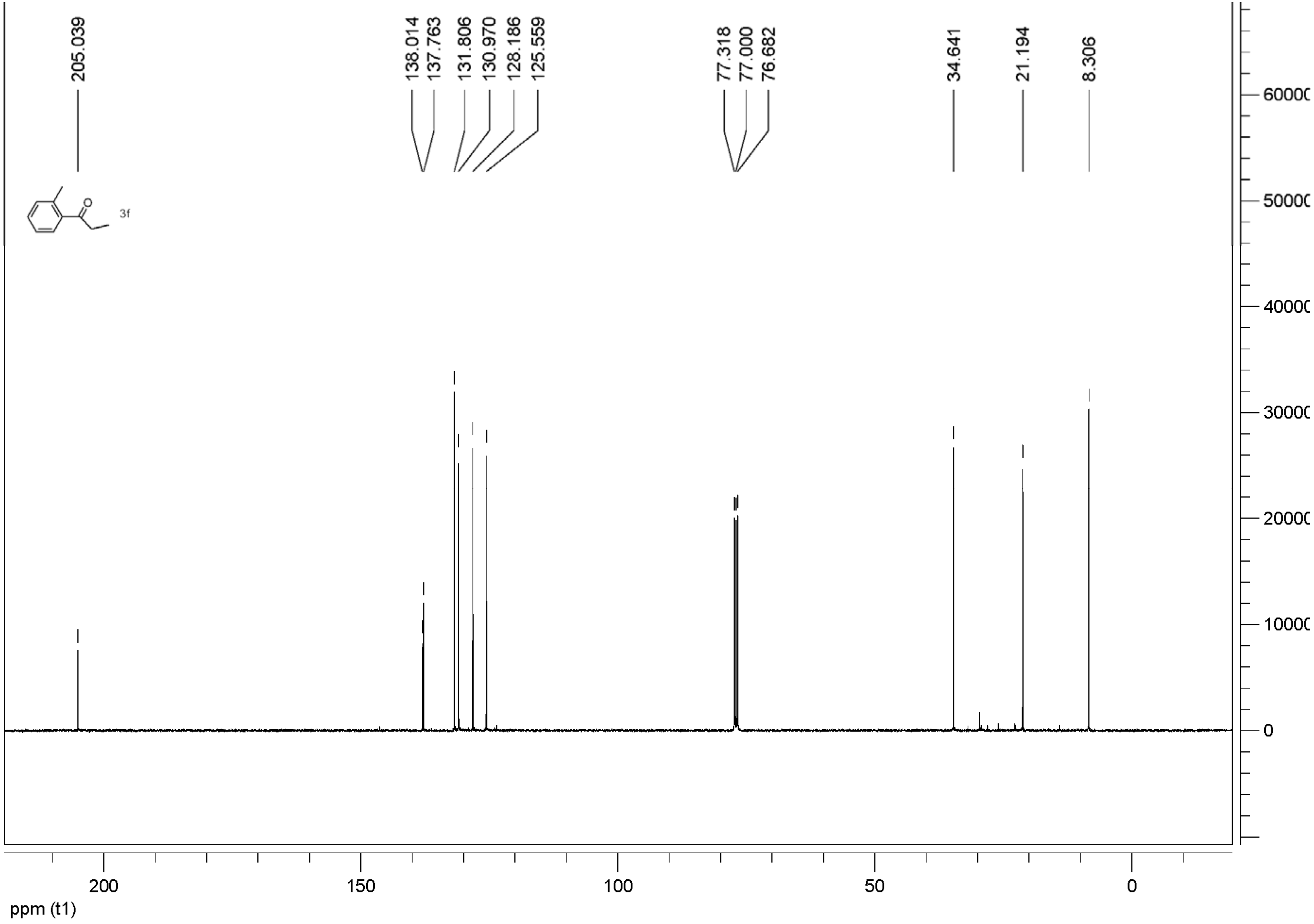

Figure S13. ^{1}H NMR of 1-phenylpentan-3-one (**3g**)

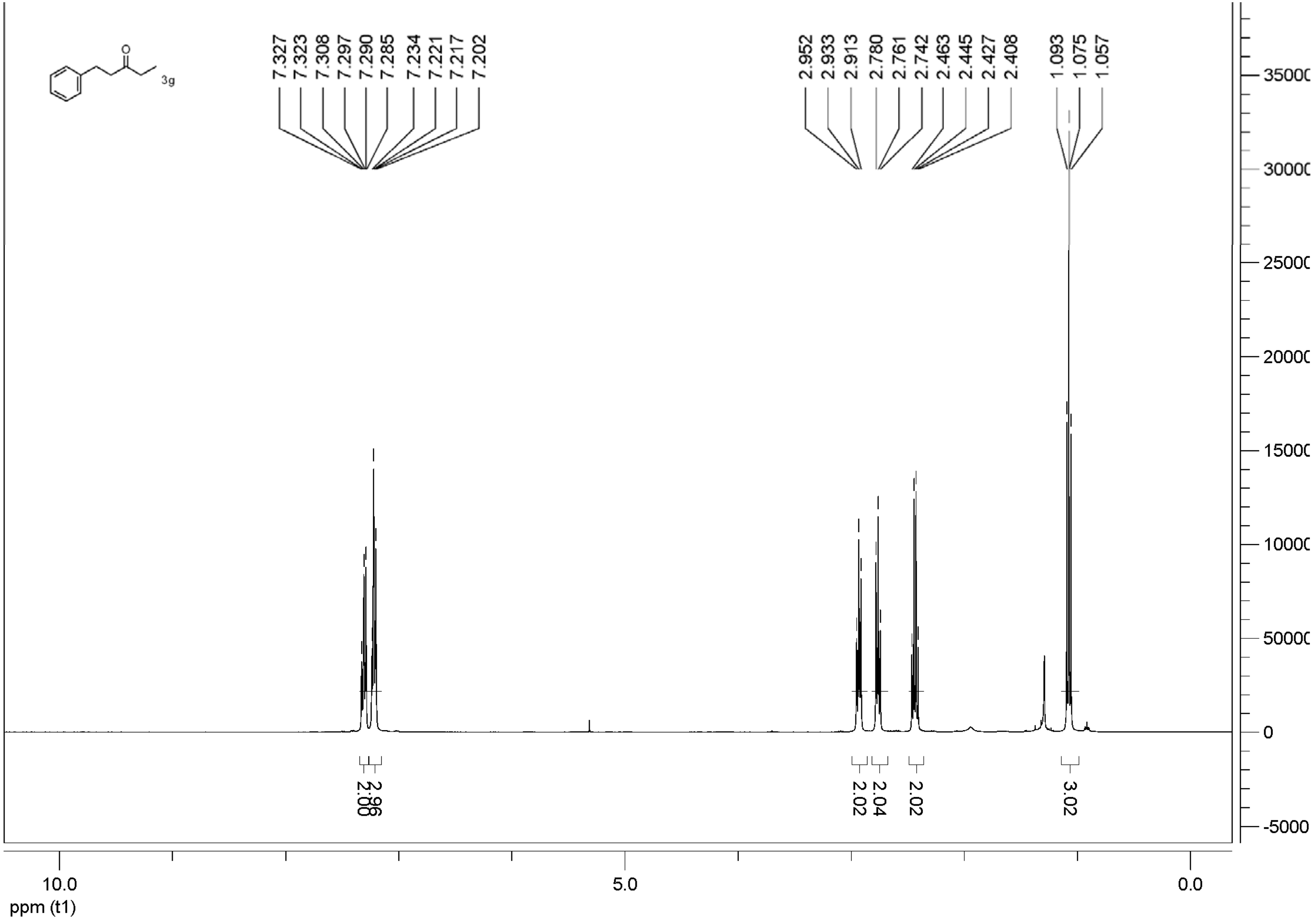

Figure S14. ^{13}C NMR of 1-phenylpentan-3-one (**3g**)

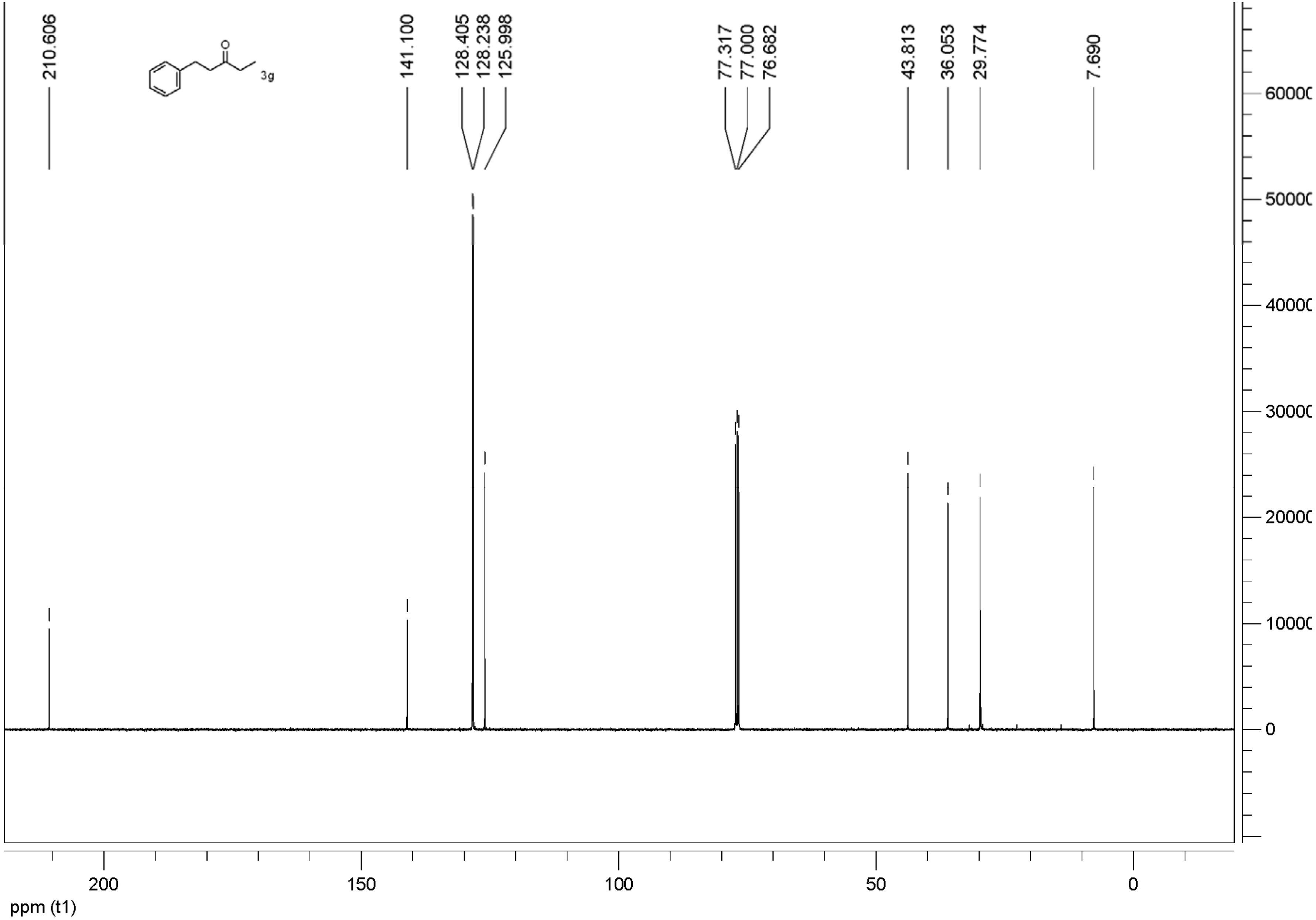

Figure S15. ^{1}H NMR of 1-(4-methoxyphenyl)pentan-3-one (**3h**)

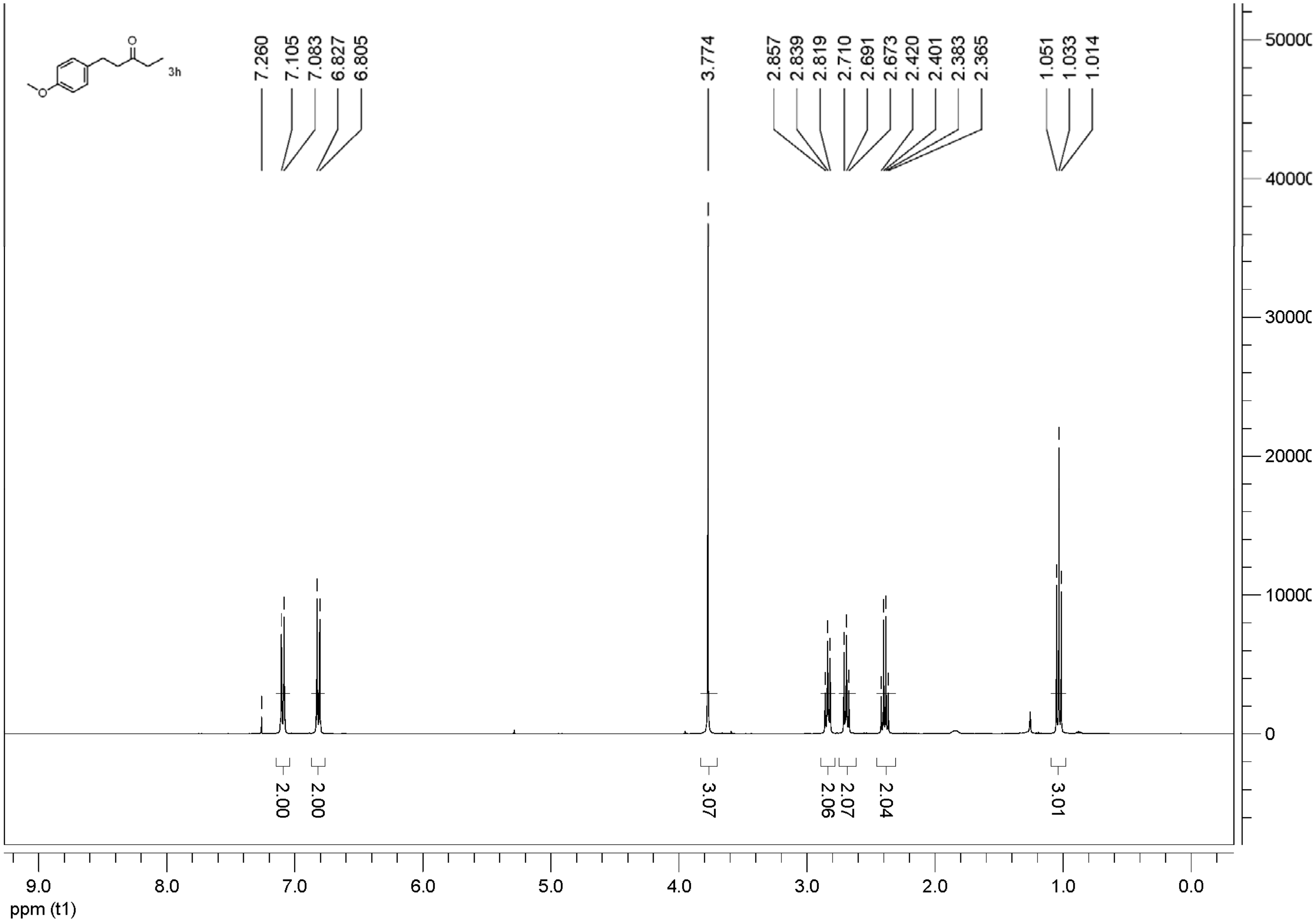

Figure S16. ^{13}C NMR of 1-(4-methoxyphenyl)pentan-3-one (**3h**)

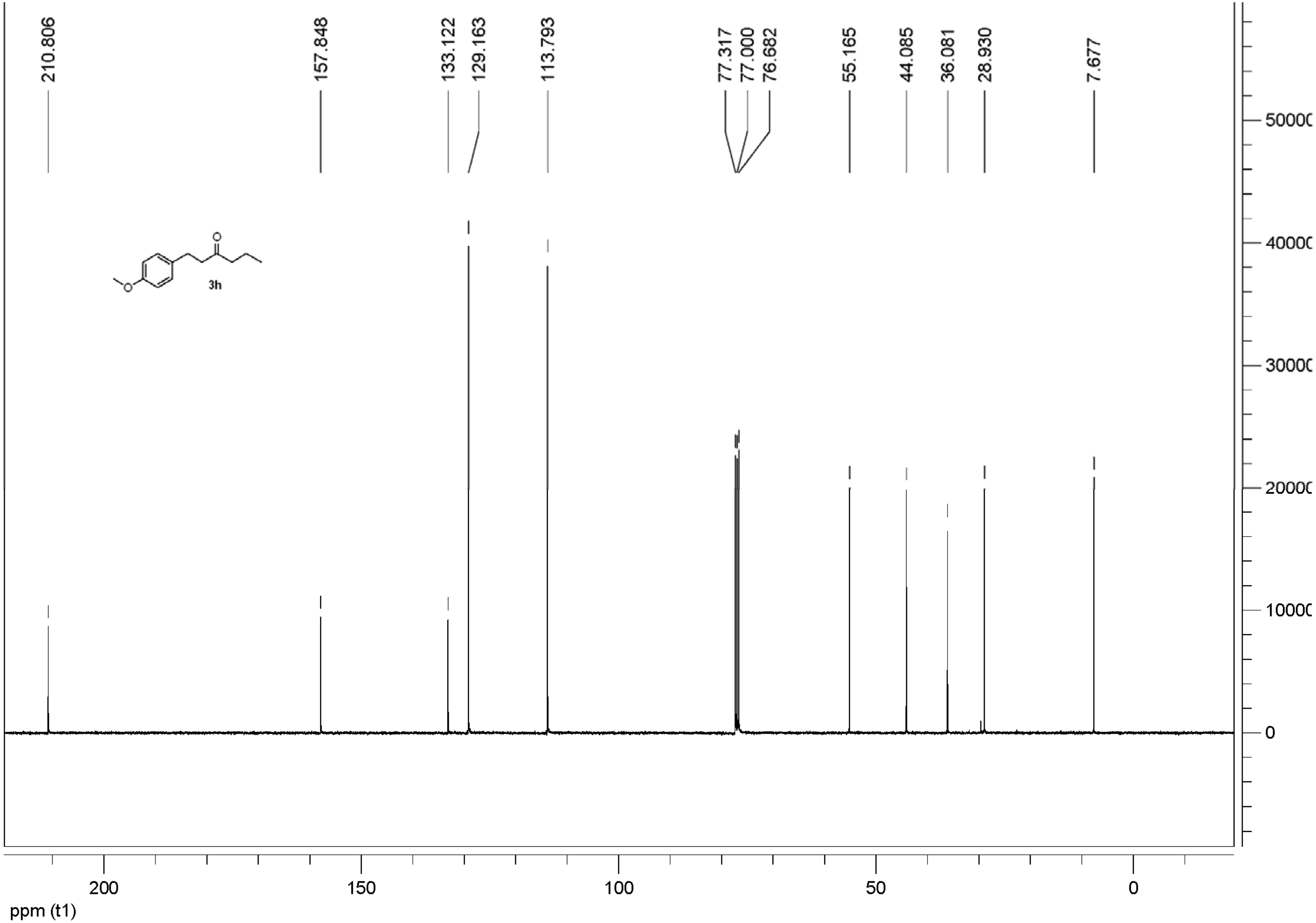

Figure S17. ^{1}H NMR of 1-(4-methoxyphenyl)pentanone (**3i**)

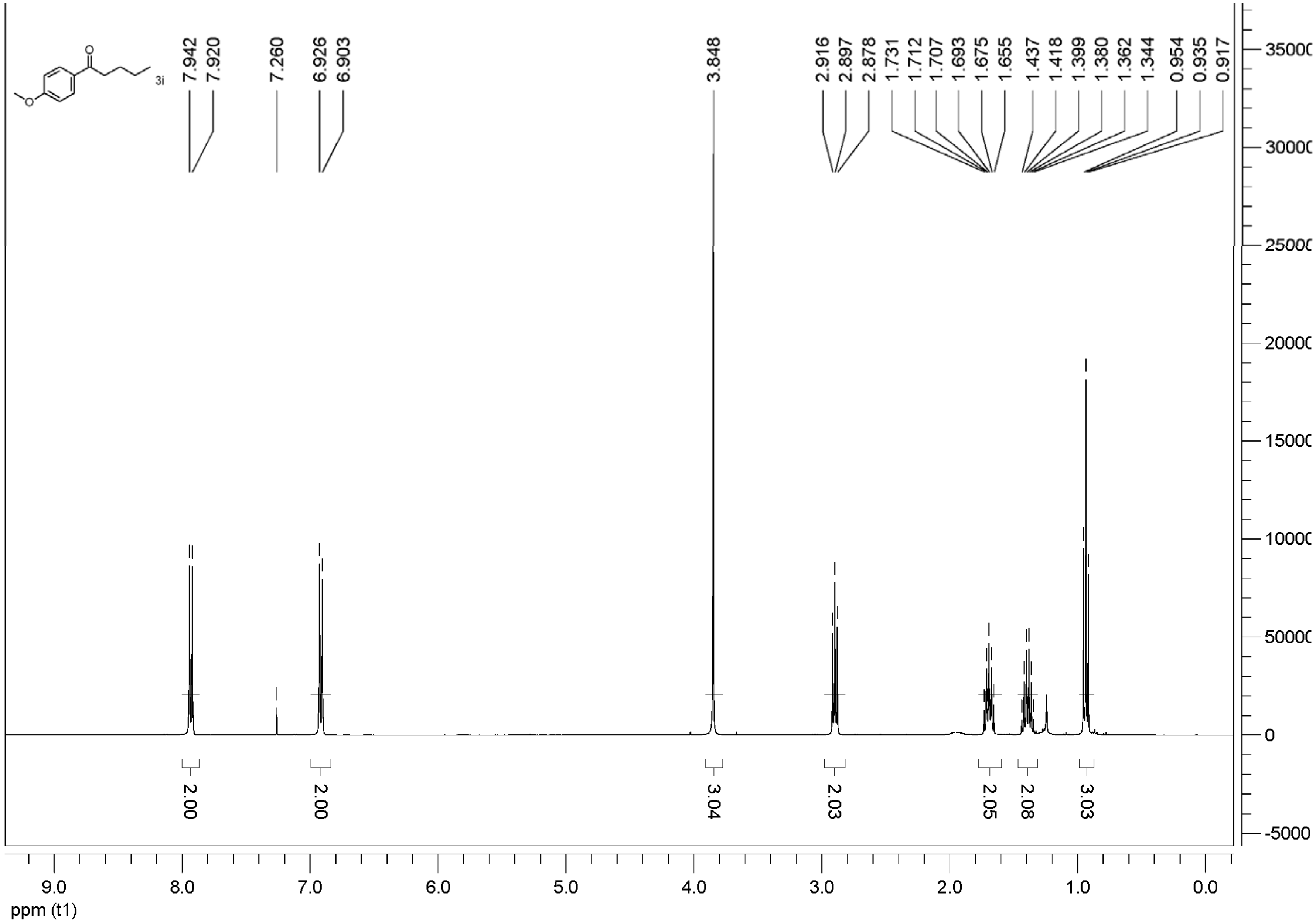

Figure S18. ^{13}C NMR of 1-(4-methoxyphenyl)pentanone (**3i**)

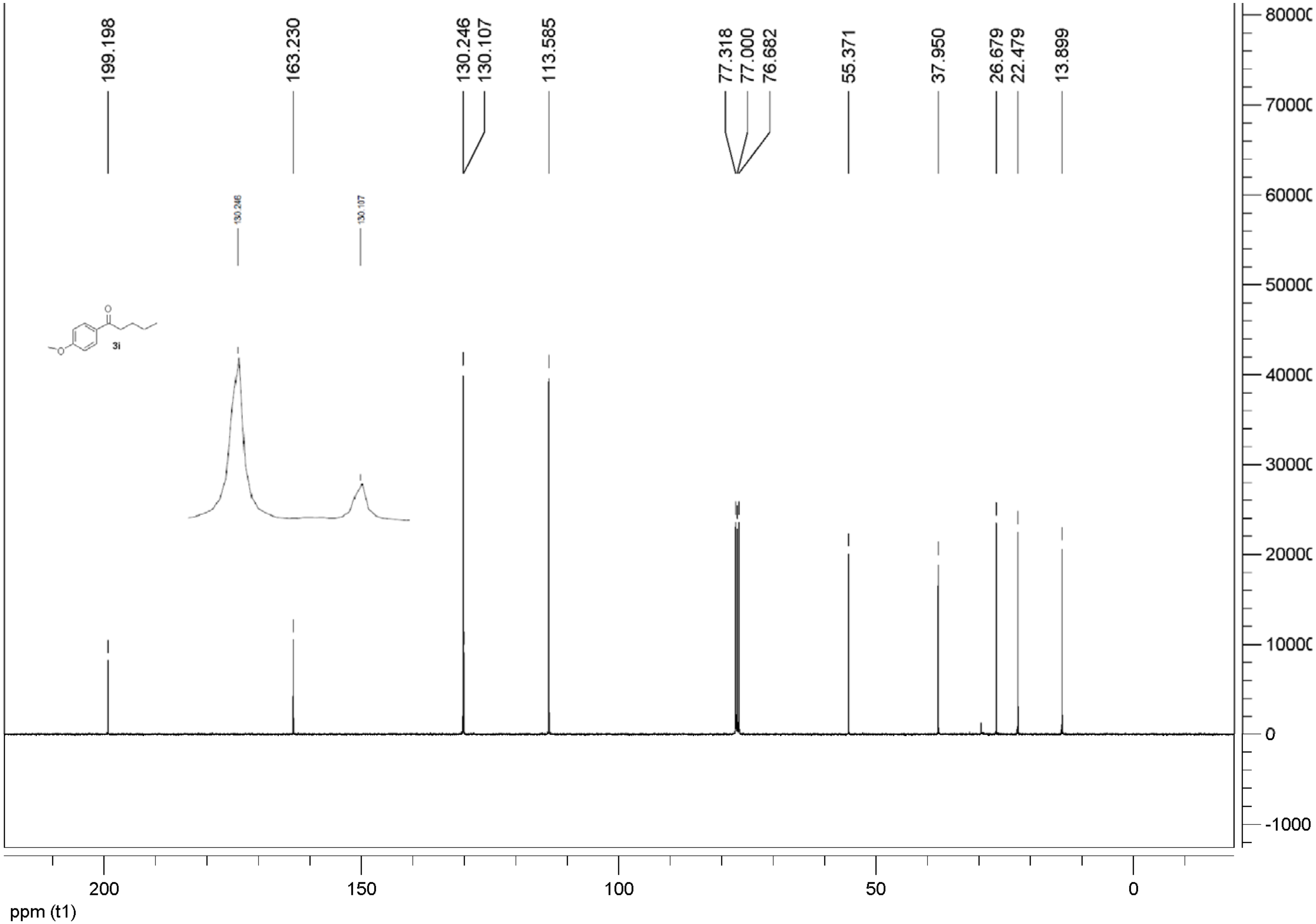

Figure S19. ^{1}H NMR of 1-(4-methoxyphenyl)nonanone (**3j**)

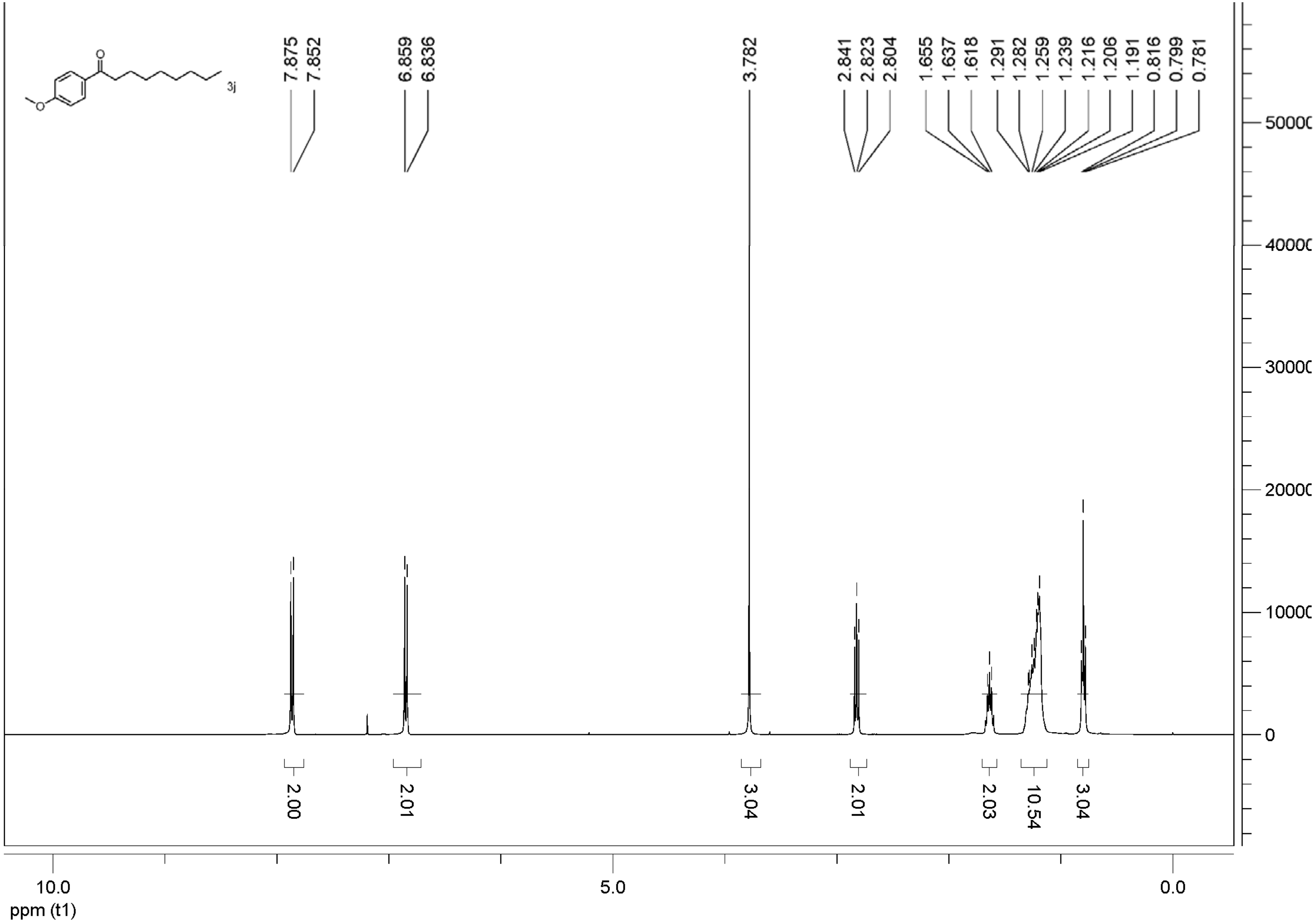

Figure S20. ^{13}C NMR of 1-(4-methoxyphenyl)nonanone (**3j**)

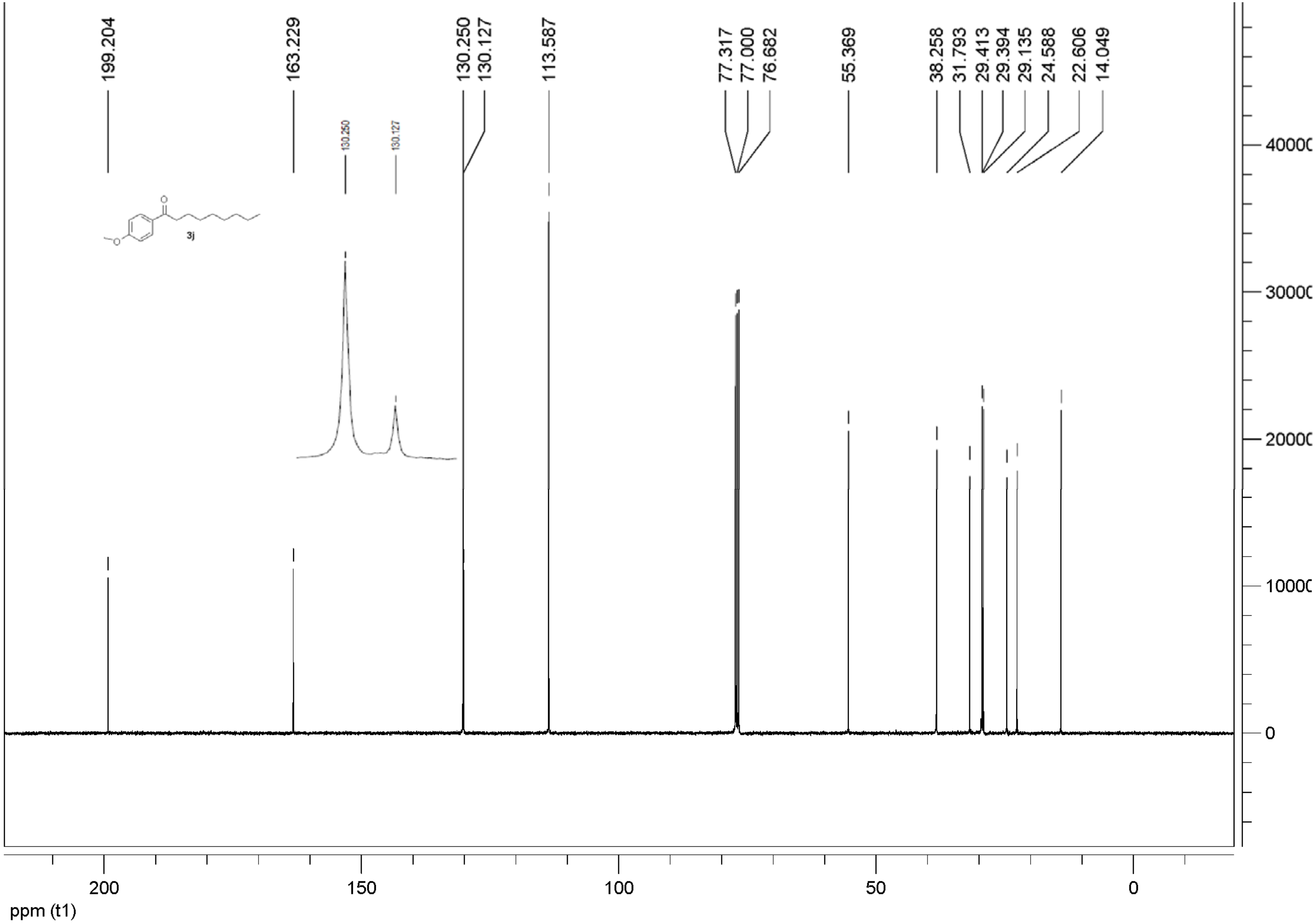

Figure S21. ^{1}H NMR of 1-(4-methoxyphenyl)-3-phenylbutanone (**3k**)

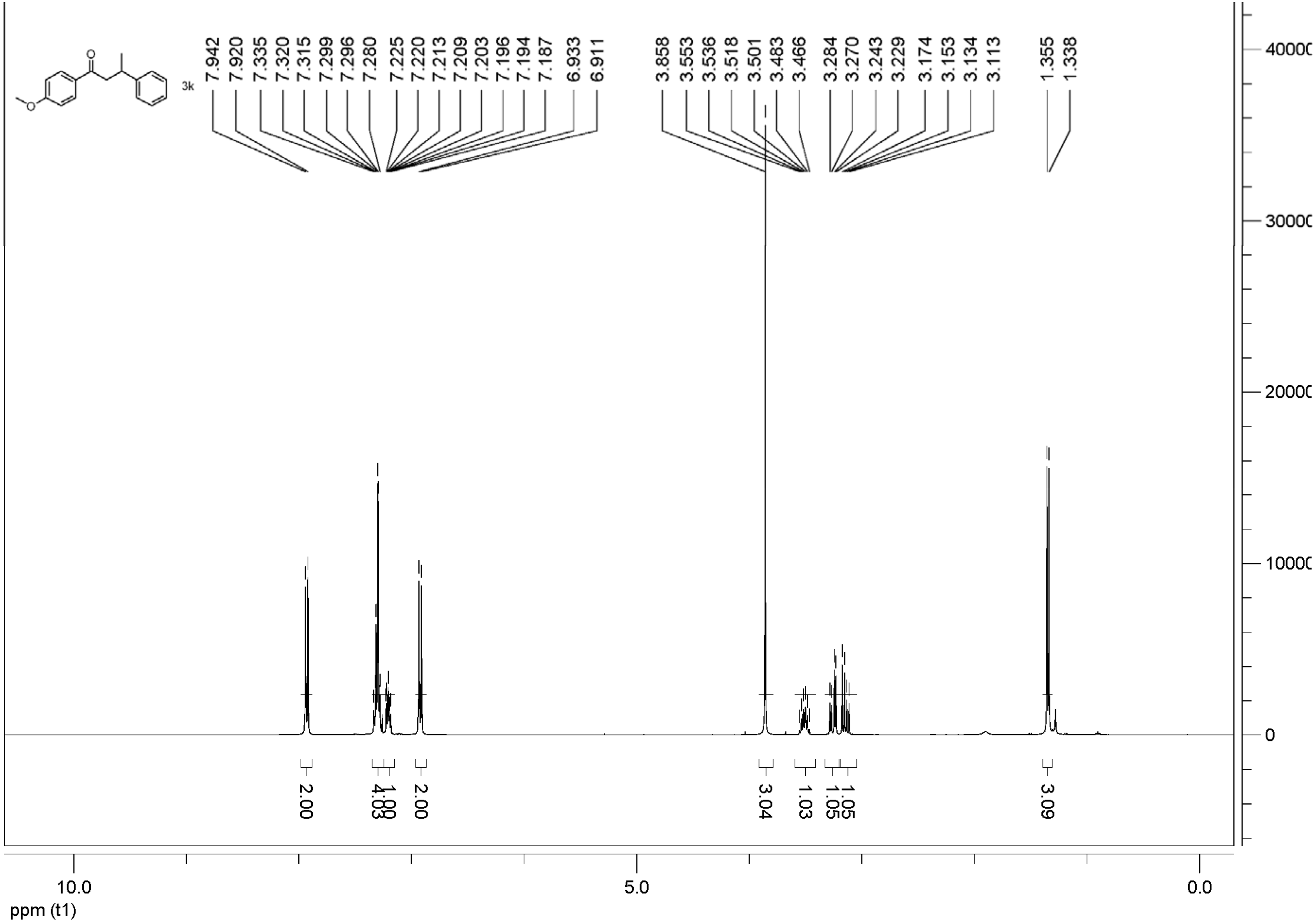

Figure S22. ^{13}C NMR of 1-(4-methoxyphenyl)-3-phenylbutanone (**3k**)

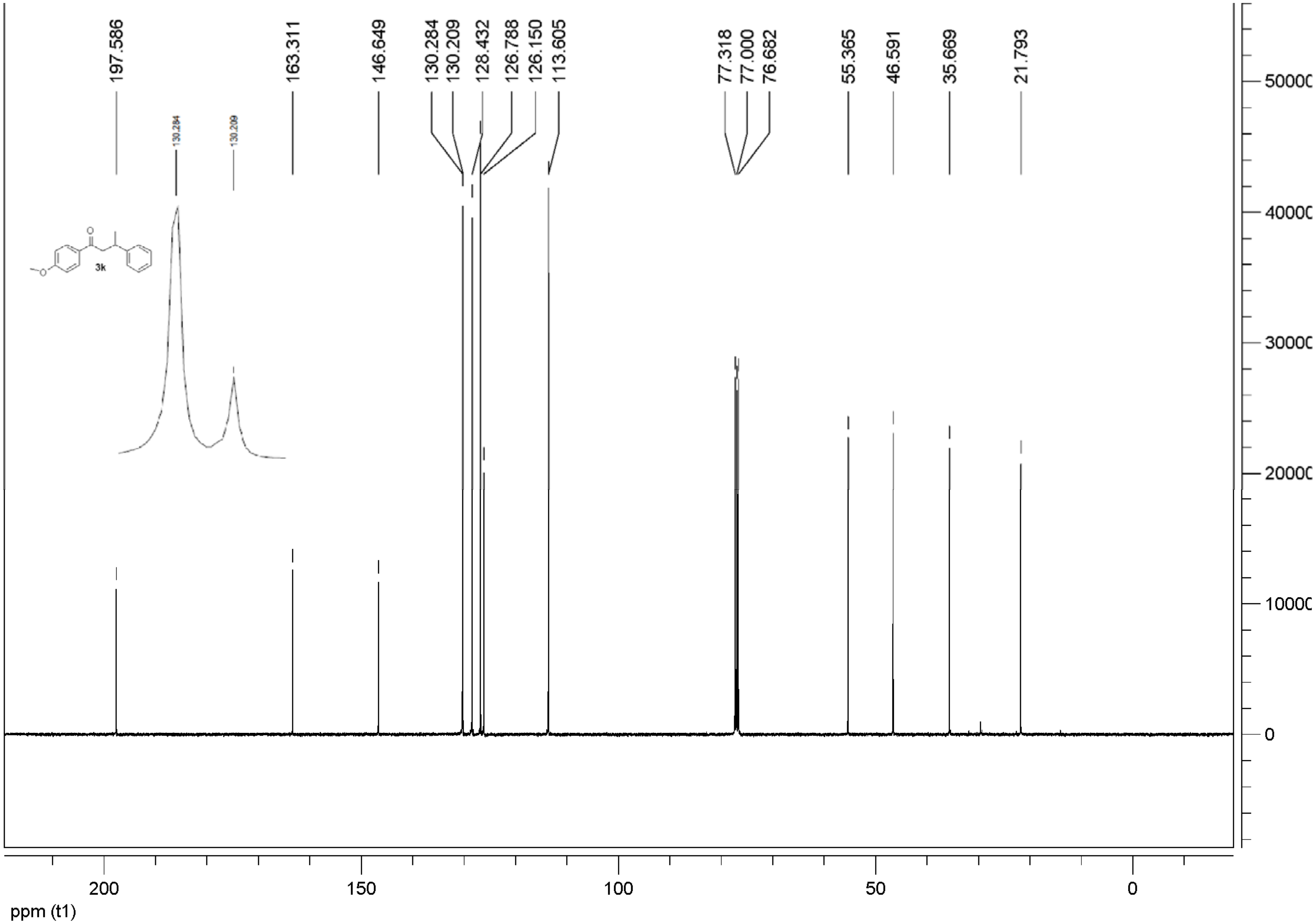

Figure S23. ^{1}H NMR of 1-cyclohexyl-3-phenylbutanone (**3l**)

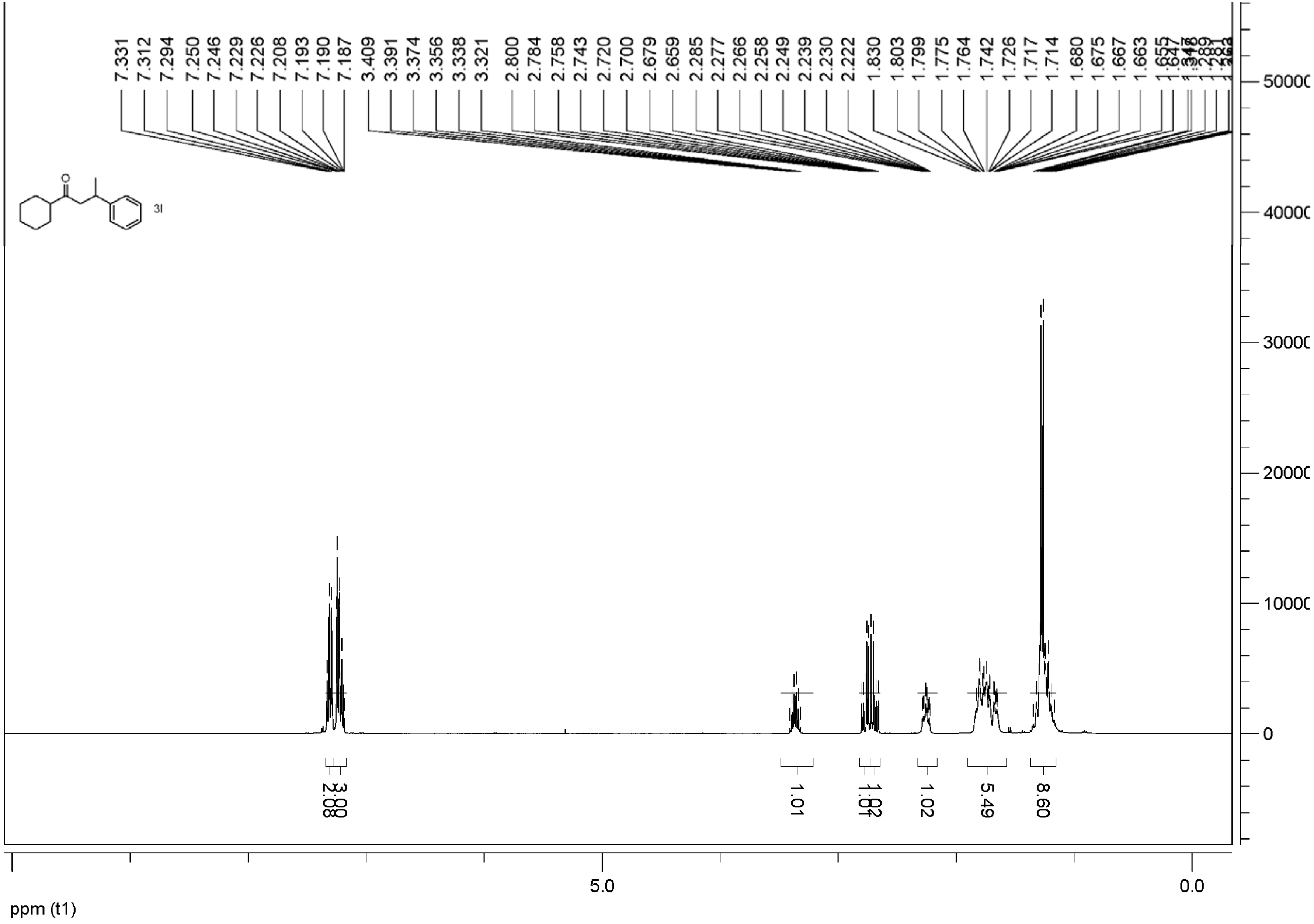

Figure S24. ^{13}C NMR of 1-cyclohexyl-3-phenylbutanone (**3l**)

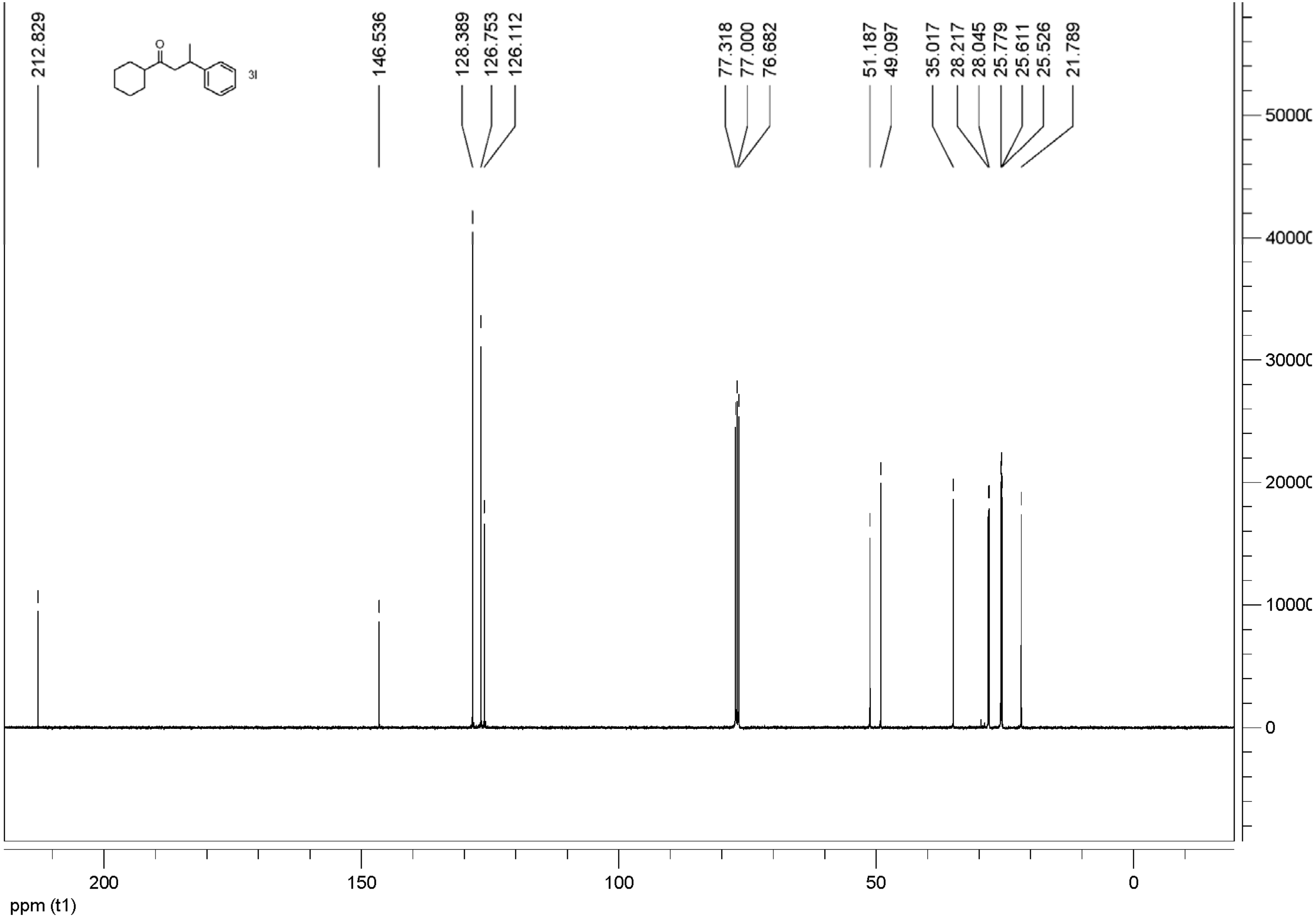

Figure S25. ^{1}H NMR of 3-phenyl-1-(thiophen-2-yl)butanone (**3m**)

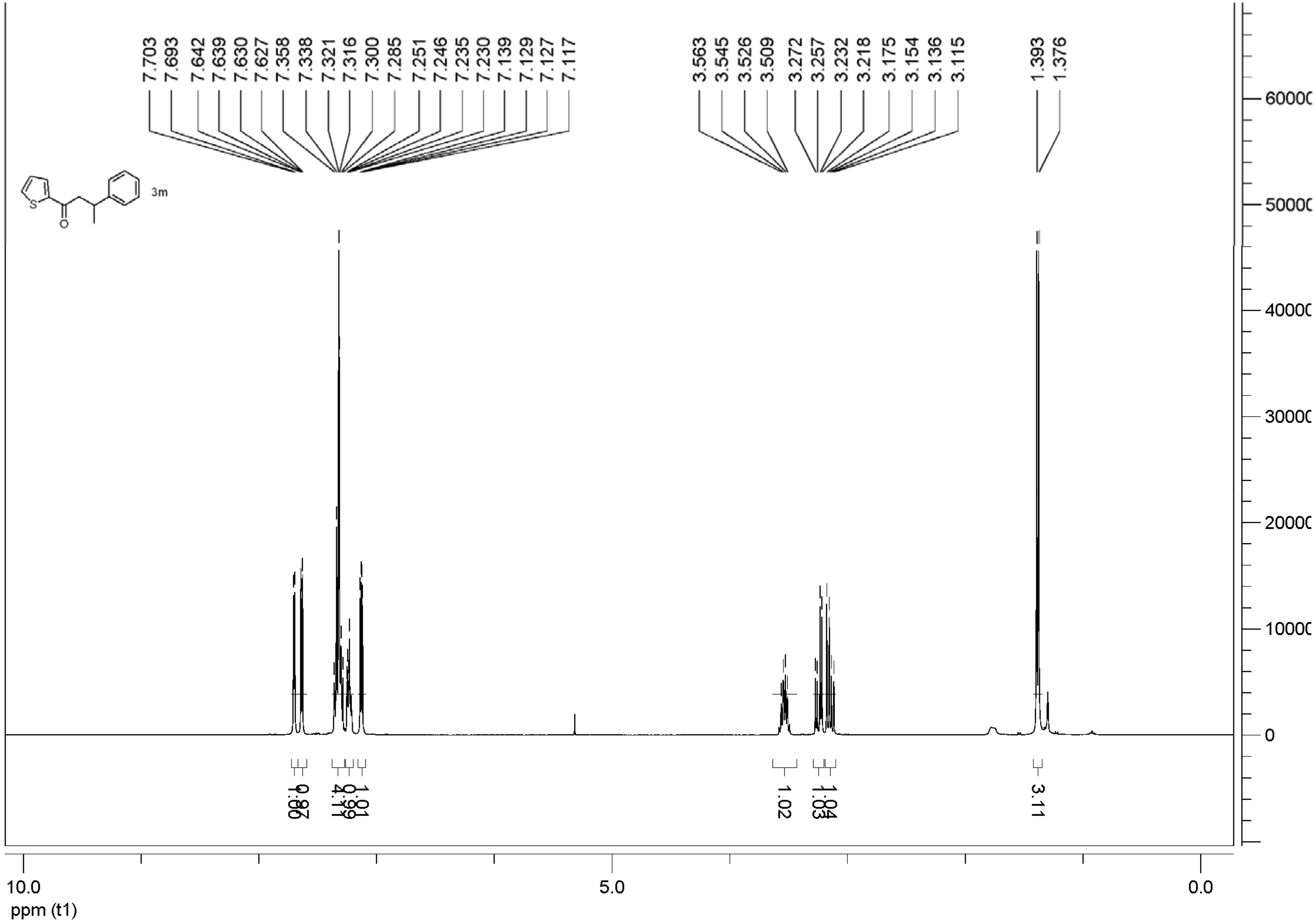

Figure S26. ^{13}C NMR of 3-phenyl-1-(thiophen-2-yl)butanone (**3m**)

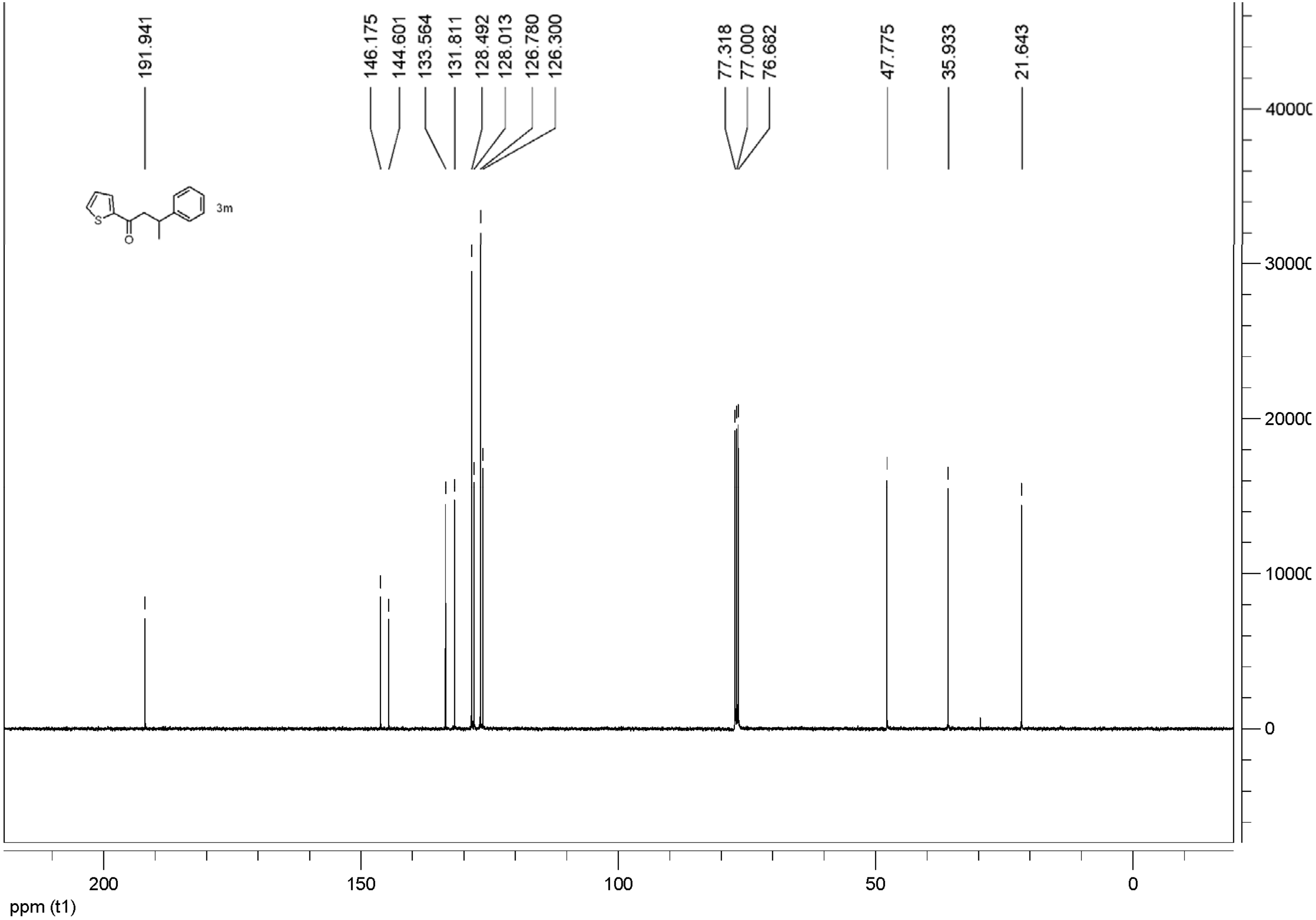

Figure S27. ^{1}H NMR of 1-phenylnonanone (**3n**)

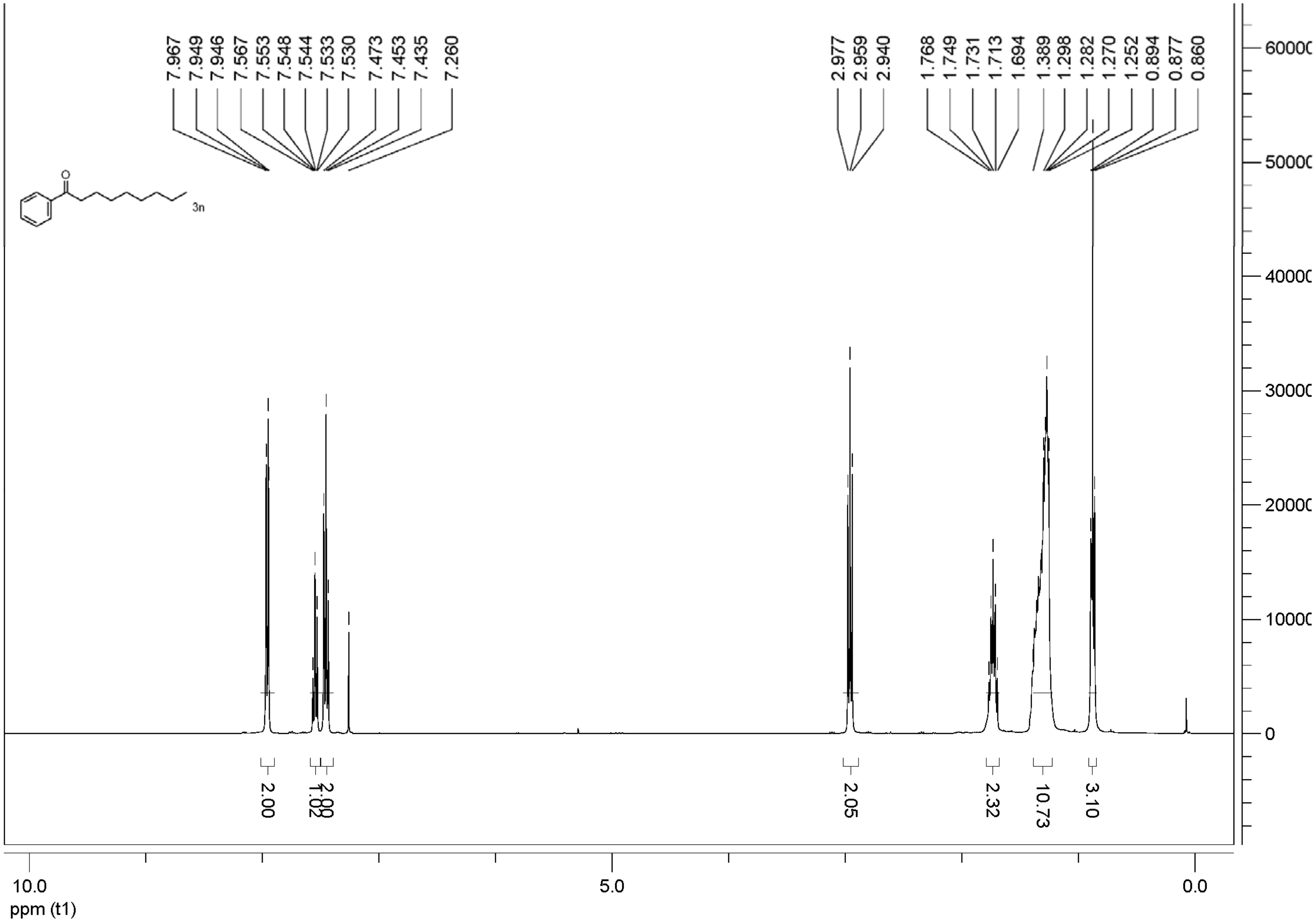

Figure S28. ^{13}C NMR of 1-phenylnonanone (**3n**)

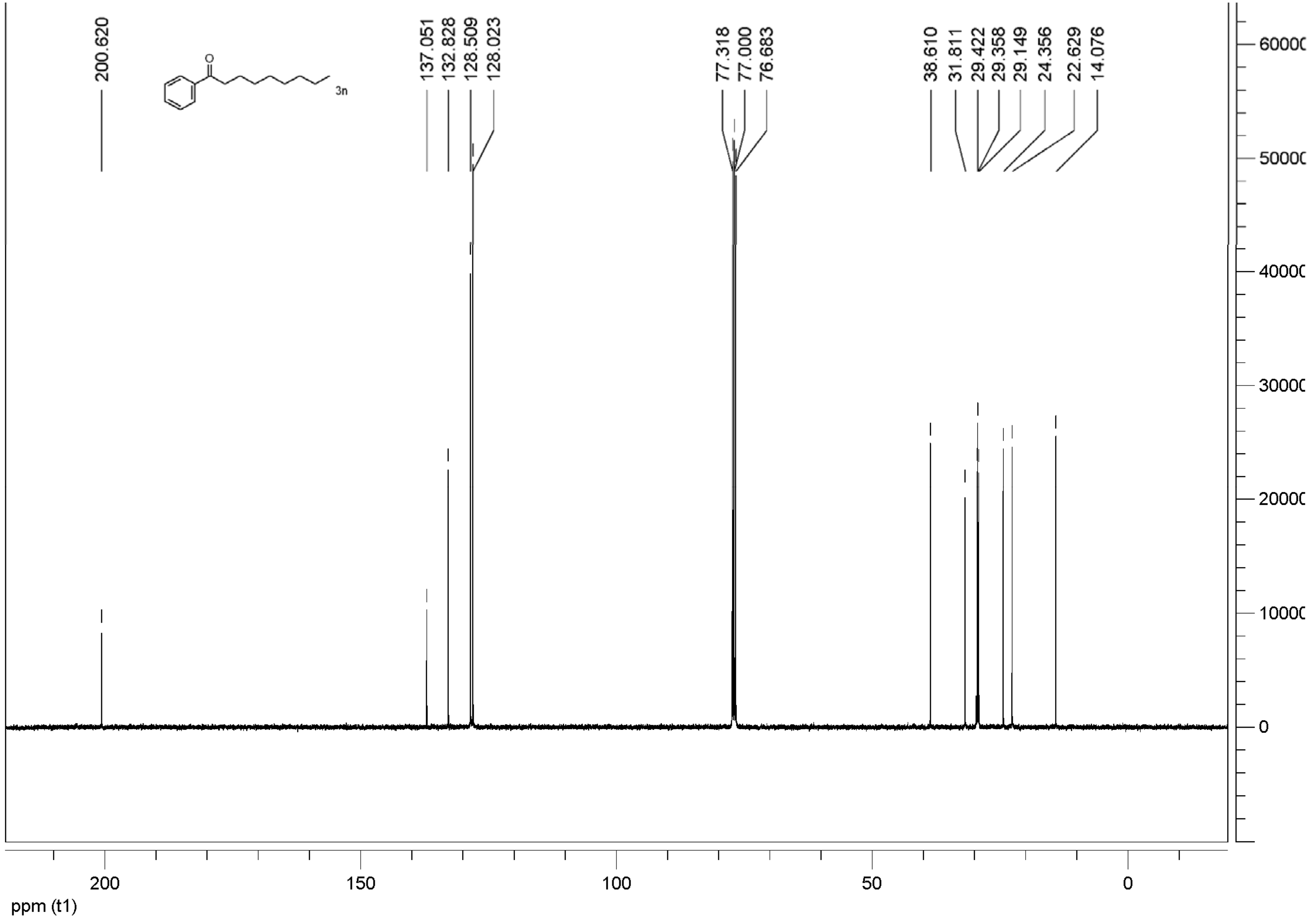

Figure S29. HRMS of 1-cyclohexyl-3-phenylbutanone (**3l**)

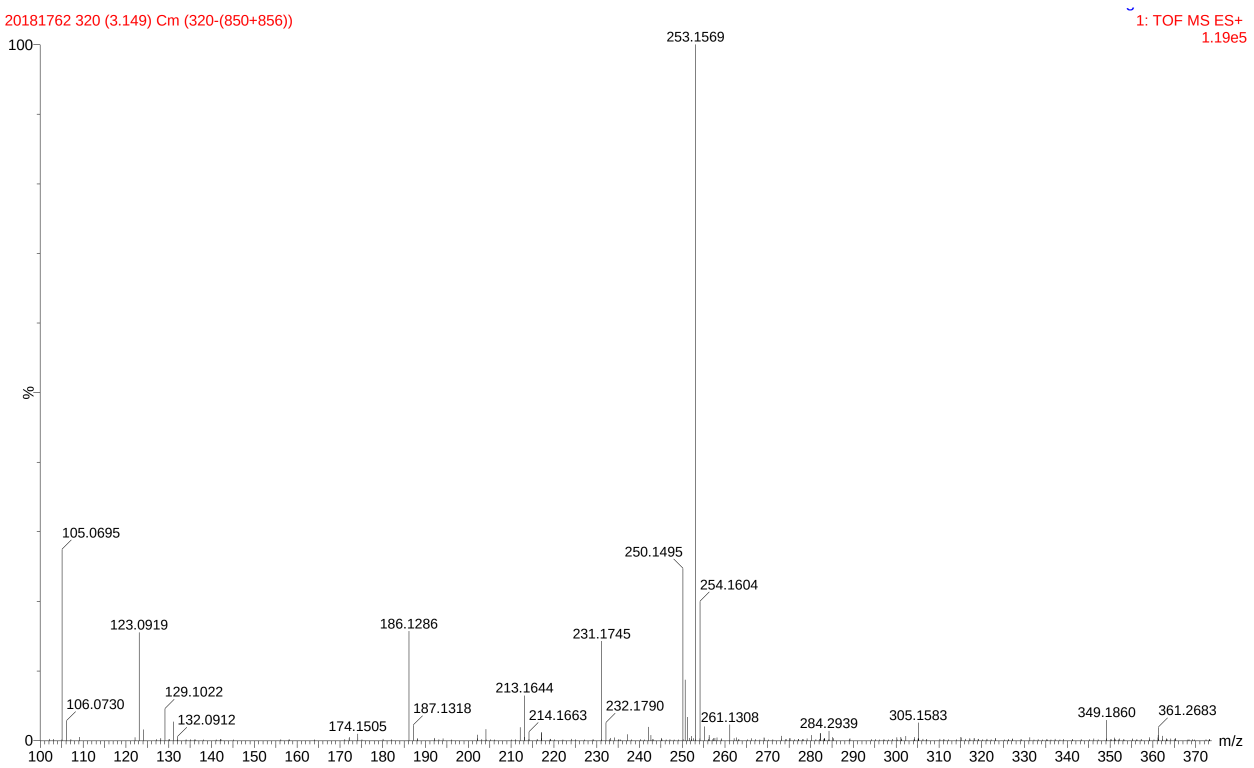

Elemental Composition Report

Multiple Mass Analysis: 2 mass(es) processed

Tolerance = 5.0 PPM / DBE: min = -1.5, max = 50.0

Element prediction: Off

Number of isotope peaks used for i-FIT = 3

Monoisotopic Mass, Even Electron Ions

808 formula(e) evaluated with 2 results within limits (all results (up to 1000) for each mass)

Elements Used:

12C: 0-50 13C: 0-1 H: 0-50 O: 0-5 Na: 0-1

Minimum:	10.00						-1.5	
Maximum:	100.00		5.0	5.0	50.0			
Mass	RA	Calc. Mass	mDa		PPM		DBE	
i-FIT	Norm	Conf(%)	Formula					
253.1569	100.00	253.1568	0.1	0.4	5.5	44.5		n/a
n/a		12C16 H22 O Na						
254.1604	19.97	254.1602	0.2	0.8	5.5	27.1		n/a
n/a		12C15 13C H22 O Na						
